# Limits of life in hostile environments: no barriers to biosphere function?

**DOI:** 10.1111/j.1462-2920.2009.02079.x

**Published:** 2009-12

**Authors:** Jim P Williams, John E Hallsworth

**Affiliations:** School of Biological Sciences, MBC, Queen's University BelfastBelfast, BT9 7BL, UK

## Abstract

Environments that are hostile to life are characterized by reduced microbial activity which results in poor soil- and plant-health, low biomass and biodiversity, and feeble ecosystem development. Whereas the functional biosphere may primarily be constrained by water activity (a_w_) the mechanism(s) by which this occurs have not been fully elucidated. Remarkably we found that, for diverse species of xerophilic fungi at a_w_ values of ≤ 0.72, water activity per se did not limit cellular function. We provide evidence that chaotropic activity determined their biotic window, and obtained mycelial growth at water activities as low as 0.647 (below that recorded for any microbial species) by addition of compounds that reduced the net chaotropicity. Unexpectedly we found that some fungi grew optimally under chaotropic conditions, providing evidence for a previously uncharacterized class of extremophilic microbes. Further studies to elucidate the way in which solute activities interact to determine the limits of life may lead to enhanced biotechnological processes, and increased productivity of agricultural and natural ecosystems in arid and semiarid regions.

## Introduction

Xerophilic fungi are more tolerant to water stress than any other organism; mycelial growth of one species has been previously recorded down to a water activity of 0.656 (Pitt and Christian, 1968). Terrestrial fungi play key roles in the degradation of organic matter and global nutrient cycles, the formation and structure of soils and geological deposits, and via their symbiotic interactions with plants (Ruiz and Azcon, 1995; Gunde-Cimerman *et al.*, 2000; Jeffries *et al.*, 2003; Hoffland *et al.*, 2004; van der Heijden *et al.*, 2008). Furthermore, the substantial fungal biomass of soils in semiarid regions (Smith *et al.*, 1992; Rutz and Kieft, 2004) can act as nutrient and water reservoirs in water-constrained ecosystems (Ruiz and Azcon, 1995; Austin *et al.*, 2004; Kashangura *et al.*, 2006; Collins *et al.*, 2008). Xerophilic microbes have historically been isolated and characterized in the context of food-spoilage studies (Pitt, 1975), but they exist in nature at an indefinite number of biosphere–environment interfaces where life is challenged by physical and chemical barriers. Xerophilic fungi are therefore useful model systems to investigate the feasibility of cellular activity in arid and stressful habitats (Onofri *et al.*, 2004; Beaty and Buxbaum, 2006; Tosca *et al.*, 2008). Recent studies carried out on halophilic prokaryotes and mesophilic bacterial and yeast species from hostile environments found that chaotropicity (or related solute activities) can limit microbial metabolism, replication and survival (Hallsworth *et al.*, 2003a; 2007; Duda *et al.*, 2004; Lo Nostro *et al.*, 2005). For both ionic and non-ionic solutes, neither chaotropic activities nor Hofmeister effects is a colligative property of a solution (see Dixit *et al.*, 2002; Ball, 2008); furthermore the mechanism of chaotropic activity for ions (see Sachs and Woolf, 2003), non-ionic solutes (see Hallsworth *et al.*, 2003a), and hydrophobic substances (McCammick *et al.*, 2009; P. Bhaganna and J.E. Hallsworth, unpublished) may differ. Nevertheless, the Hofmeister series (for ions), chaotropicity and kosmotropicity (activities of diverse chemical species) provide frameworks that can be usefully employed to study the affinities of substances to modify the structural interactions of cellular macromolecules (see Hamaguchi and Geiduschek, 1962; Collins, 1997; Hallsworth *et al.*, 2003a; Ball, 2008). However, such solute activities have not been studied either at ultra-low water activities (≤ 0.8; see Hallsworth *et al.*, 2007), or in xerophilic fungi. Water activity has been highly effective in providing a global measure of the cumulative molecular-level, biochemical and phenotypic effects of decreased solvent availability (see Brown, 1990; Chaplin, 2006). Nevertheless, we recently observed that other solutes activities, most notably chaotropicity that weakens macromolecular interactions and disorders cellular structures, can also limit biosphere function in specific localities (see Hallsworth *et al.*, 2007). We therefore suspected that water activity is not a definitive parameter that dictates the limits of microbial activity in all environmental niches. We carried out this study of xerophiles, obtained from diverse sources, to test the hypothesis that water activity does not always act as the barrier to microbial and, by implication, biosphere function in high-solute environments. Here we show that in low water-activity environments that are hostile to life (≤ 0.72 a_w_), water activity per se did not limit microbial activity; and provide evidence that cellular function was determined by the net effect of other environmental parameters (including chaotropic and kosmotropic activities) that impact on macromolecule structure-function. We also identified a new class of extremophilic microbe, *chaophiles*, that may prove to be a source of novel enzymes for biotechnology.

## Results and discussion

### Xerophilic fungi from high- and low-solute substrates

We took a two-pronged approach to identifying the ultimate, most xerophilic, microbes. First, we sampled environments in various continents and climatic zones, focusing our search on low-solute substrates: the surfaces of glass, metal, wood, leather, textiles and paper (see Table 1; *Experimental procedures*). Remarkably we found an abundance of xerophilic fungi, and isolated 107 phenotypically distinct cultures from these low-solute environments using glycerol-supplemented (5 M glycerol; 0.845 a_w_) or sucrose-supplemented media (2.2 M sucrose; 0.884 a_w_), predominantly on samples originating from humid countries such as Japan, Northern Ireland and Thailand (see Table 1). Second, we identified and obtained cultures of the 37 most-xerophilic strains previously reported in the published literature (1900–2008); the majority of these had been isolated from high-solute foods (see Table 2). In addition, we contacted research groups currently working in the field of environmental microbiology and obtained cultures of fungi and yeasts that had been isolated from high-salt or high-sugar environments and/or were suspected to be highly solute-tolerant (i.e. the 14 strains from EXF and UWOPS culture collections; see Table 2). For the purposes of the current study we used multiple criteria to define xerophilicity: that a species must be able to grow below 0.85 a_w_ under at least two sets of environmental conditions, and must also grow optimally below 0.95 a_w_ (see Pitt, 1975).

**Table 2:** Named xerophilic and solute-tolerant species that were used in the current study.^a^

Species	Strain designation	Environmental source (country)	Relevant reference(s)
*Aspergilus glaucus*	IMI 053242	Microscope objective (Sri Lanka)	
*Aspergilus nidulans var. echinulatus*	CBS 120.55; IMI 061454ii	Not stated (Argentina)	Fennell and Raper (1955)
*Aspergilus penicillioides*	ATTC 14567; FRR 3735	Binocular lens (Australia)	
*A. penicillioides*	ATTC 16910; FRR 3722	Human lobomycosis (Australia)	Gock *et al.* (2003)
*A. penicillioides*	FRR 2179	Dried chillies (Australia)	
*A. penicillioides*	FRR 3795	Audio tape (Australia)	
*Aspergillus wentii*	CBS 104.07; IMI 017295ii	Soybeans (Indonesia)	
*Basipetospora chlamdospora*	IMI 332258	Soil (Chile)	
*Brettanomyces bruxellensis*	UWOPS 94-239.3	Tequila fermentation (Mexico)	
*Candida apicola*	UWOPS 01-663b2	*Merremia tuberosa* flower (Costa Rica)	
*Candida berthetii*	ATCC 18808; CBS 5452	Arabic gum (Cameroon)	Boidin *et al.* (1963)
*Candida etchellsii*	UWOPS 01-168.3	Bee hive (Costa Rica)	
*Candida hawaiiana*	UWOPS 04-206.8	*Prosopeus cf. bidens* (nitidulid beetle) from *llex anomala* flower (Hawaii)	
*Chrysosporium fastidium*	ATTC 18053; FRR 0077	Improperly sundried prunes (Australia)	Hocking and Pitt (1980); Pitt and Hocking (1977)
*C. fastidium*	FRR 0081	Dried prunes (Australia)	
*Chrysosporium xerophilium*	ATTC 18052; FRR 0530	High-moisture prunes (Australia)	Kinderlerer (1995)
*Cladosporium sphaerospermum*	EXF 738	Bathroom (Slovenia)	Zalar *et al.* (2007)
*Debaryomyces hansenii*	DSMZ 3428	Spoilt sake (not stated)	
*D. hansenii*	DSMZ 70590	Harzer cheese	
*D. hansenii*	UWOPS 05-230.3	Beetle, Bertam Palm (Malaysia)	
*Debaryomyces melissophilus*	UWOPS 01-677c6	*Conotelus* (nitidulid beetle) from *M. tuberosa* flower (Costa Rica)	
*Eurotium amstelodami*	ATTC 16464; FRR 2792	Dates (Australia)	Tamura *et al.* (1999)
*E. amstelodami*	ATTC 42685; FRR 0475	Dried prunes (Australia)	Hocking and Pitt (1980)
*Eurotium chevalieri*	ATTC 28248; FRR 1311	Spoiled prunes (Australia)	Pitt and Hocking (1977)
*Eurotium echinulatum*	FRR 2419	Hazelnut kernels (Australia)	
*E. echinulatum*	FRR 5040	Sultanas (Australia)	
*Eurotium halophilicum*	ATTC 62923; FRR 2471	Cardamom seeds (Australia)	Hocking and Pitt (1988)
*Eurotium herbariorum*	FRR 2418	Hazelnut kernels (Australia)	
*E. herbariorum*	FRR 5004	Sultanas (Australia)	
*E. herbariorum*	FRR 5354	Liquorice (Australia)	
*Hortaea werneckii*	EXF 225	Hypersaline saltern (Slovenia)	
*Kodamaea ohmeri*	UWOPS 05-228.2	Beetle, Bertam Palm (Malaysia)	
*Pichia sydowiorum*	UWOPS 03-414.2	Nectar, Bertam Palm (Malaysia)	
*Polypaecilum pisce*	FRR 2732; IMI 288726ii	Dried fish (Indonesia)	
*Saccharomyces cerevisae*	CCY 21-4-13	Not stated (not stated)	
*Saccharomyces ludwigii*	UWOPS 92-218.4	Tequila fermentation (Mexico)	
*Starmerella bombicola*	UWOPS 01-123.1	Bee from *Ipomoea trifida* (Costa Rica)	
*Wallemia ichthyophaga*	CBS 818.96	Sunflower seed (Sweden)	Vaupotic and Plemenitas (2007); Zalar *et al.* (2005)
*Wallemia muriae*	MZKI B-952	Hypersaline saltern (Slovenia)	Vaupotic and Plemenitas (2007); Zalar *et al.* (2005)
*Wallemia sebi*	EXF 994	Hypersaline saltern (Slovenia)	Zalar *et al.* (2005)
*W. sebi*	EXF 1053	Dead Sea (Israel)	Zalar *et al.* (2005)
*W. sebi*	FRR 4623	Maple syrup (Australia)	
*Xeromyces bisporus*	ATTC 28298; FRR 0025	High-moisture prunes (Australia)	
*X. bisporus*	ATTC 36964; FRR 1522	Spoiled liquorice (Australia)	Hocking and Pitt (1980); Pitt and Hocking (1977)
*X. bisporus*	FRR 2347	Fruit cake (Australia)	Gock *et al.* (2003)
*X. bisporus*	FRR 3443	Raisins (Australia)	
*X. bisporus*	IMI 317902	Chinese dates (Australia)	
*Zygosaccharomyces rouxii*	ATTC 28166; FRR 3669	Table wine (Australia)	Andrews and Pitt (1987)
*Z. rouxii*	FRR 3681	Fructose corn-syrup (Australia)	Andrews and Pitt (1987)
*Z. rouxii*	FRR 5304; NCYC 381	Sugarcane (Australia)	Corry (1976)

a Cultures were obtained from the American Type Culture Collection (ATTC, USA), the Centraalbureau voor Schimmelcultures (CBS, Netherlands), the Culture Collection of Yeasts (CCY, Slovakia), the German Collection of Microorganisms and Cell Cultures (DSMZ, Germany), the Extremophilic Fungi Culture Collection (EXF, Slovenia), the Food Research Ryde (FRR, Australia), the International Mycological Institute (IMI, UK), the Microbial Culture Collection of National Institute of Chemistry (MZKI, Slovenia), the National Collection of Yeast Cultures (NCYC, UK), and the University of Western Ontario Plant Sciences Culture Collection (UWOPS, Canada).

**Table 1:** Fungal strains isolated from diverse substrates during the current study.^a^

Strain designation^b^	Environmental source (country)	Strain designation^b^	Environmental source (country)
JH05GB42	Copper pipe in 12°C constant-temperature room (UK)	JW07JP14	Dead bamboo (Japan)
JH05GB43	Copper pipe in 12°C constant-temperature room (UK)	JW07JP18	Surface of firewood in outdoor woodpile (Japan)
JH06GBa	Underside of an antique earthenware-bowl (UK)	JW07JP20	External wall of a wooden hut (Japan)
JH06GBb	Dust on the floor of a living room (UK)	JW07JP21	Insect pupa (Japan)
JH06GBc	Blue (Stilton) cheese (UK)	JW07JP25	Surface of firewood in outdoor woodpile (Japan)
JH06GBB	Stem of dried protea flower (South Africa)	JW07JP29	Aluminium windowsill on the outside of a building (Japan)
JH06GBl	Paint work of a 1922 wooden window-frame (UK)	JW07JP30a	Aluminium windowsill inside a building (Japan)
**JH06GBM**	**Underside of an antique sycamore chopping-block (UK)**	JW07JP30b	Aluminium windowsill inside a building (Japan)
JH06GBN	Underside of an antique sycamore chopping-block (UK)	JW07JP30c	Aluminium windowsill inside a building (Japan)
**JH06GBO**	**Underside of an antique sycamore chopping-block (UK)**	JW07JP36	Glass surface of a window inside a building (Japan)
JH06GBW	Antique felt (UK)	JW07JP41a	Wooden floor (Japan)
JH06IL49	Semi-dried date (Israel)	JW07JP41b	Wooden floor (Japan)
JH06IL50	Semi-dried date (Israel)	JW07JP43	Old glass light-bulb (Japan)
JH06IN45	Semi-dried tamarind pods (India)	JW07JP49	Underside of a stone table – outdoors (Japan)
JH06IN46	Semi-dried tamarind pods (India)	JW07JP51	Surface of wooden bench – outdoors (Japan)
JH06IN47	Antique wooden artefact (India)	JW07JP61	Rotting wood (Japan)
JH06IN48	Antique wooden artefact (India)	JW07JP64	Dead tree-trunk (Japan)
JH06JPj	Antique wooden artefact (Japan)	JW07JP74	Aluminium windowsill inside a building (Japan)
**JH06JPD**	**Antique wooden rice-scoop (Japan)**	JW07JP75	Old cotton cushion-cover (Japan)
JH06JPE	Inner surface of an antique bronze bell (Japan)	JW07JP83	Tree trunk (Japan)
JH06JPF	Inner surface of an antique bronze bell (Japan)	JW07JP95	Surface of wooden bench – outdoors (Japan)
JH06JPQ	Antique wooden rice-pot lid (Japan)	JW07JP96	Stone table – outdoors (Japan)
JH06JPS	Antique wooden rice-pot lid (Japan)	JW07JP99	Underside of a wooden bench – outdoors (Japan)
JH06JPT	Antique wooden rice-pot lid (Japan)	JW07JP117a	Internal surface of dried bamboo (Japan)
JH06NAV	Stem of a wild grape (Namibia)	JW07JP117b	Internal surface of dried bamboo (Japan)
**JH06THH**	**Antique wooden artefact (Thailand)**	JW07JP120a	Antique wooden artefact (Japan)
JH06THI	Antique wooden artefact (Thailand)	JW07JP120b	Antique wooden artefact (Japan)
**JH06THJ**	**Antique wooden artefact (Thailand)**	JW07JP160	Antique wooden artefact (Japan)
JH06THK	Antique wooden artefact (Thailand)	JW07JP166	Rotting bamboo (Japan)
JH06ZA44	Grass basket (South Africa)	JW07JP167	Rotting bamboo (Japan)
JH06ZA51	Tin surface of a food can (South Africa)	JW07JP168a	Rotting bamboo (Japan)
JH06ZA52	Tin surface of a food can (South Africa)	JW07JP168b	Rotting bamboo (Japan)
JH06ZAU	Glass of a 1940's picture frame (South Africa)	JW07JP169	Rotting bamboo (Japan)
JH07JP126	Antique bronze vase (Japan)	JW07JP170a	Rotting bamboo (Japan)
JH07JP127	Green leaf (Japan)	JW07JP170b	Rotting bamboo (Japan)
JH07JP128	Old earthenware bonsai-container (Japan)	JW07JP171a	Rotting bamboo (Japan)
JH07JP130	Green bamboo (Japan)	JW07JP171b	Rotting bamboo (Japan)
JH07JP133	Rotting wood (Japan)	JW07JP172	Rotting bamboo (Japan)
JH07JP138	Old cedarwood-container (Japan)	JW07JP173	Old, dried Reiki mushroom (Japan)
JH07JP141	Bamboo leaf (Japan)	JW07JP174	Old, dried Reiki mushroom (Japan)
JH07JP143	Green bamboo (Japan)	JW07JP175a	Old, dried Reiki mushroom (Japan)
JH07JP144	Leaf surface (Japan)	JW07JP175b	Old, dried Reiki mushroom (Japan)
JH07JP146	Dead bamboo (Japan)	JW07JP176	Old, dried Reiki mushroom (Japan)
JH07JP148	Rotting bamboo (Japan)	JW07JP177	Old, dried Reiki mushroom (Japan)
JH07JP149	Rotting bamboo (Japan)	JW07JP179	Old, dried Reiki mushroom (Japan)
JH07JP151	Rotting leaf (Japan)	JW07JP180	Moulding surface of tree branch (Japan)
JH07JP154	Wooden bathroom wall (Japan)	JW07JP181	Moulding surface of bamboo (Japan)
JH07JP156	Wooden bathroom wall (Japan)	JW07JPc118	Airborne spores (Japan)
JH07ZA147	Wooden artefact (South Africa)	RS07PT1	Laboratory contaminant (Portugal)
JW07GB158	Antique mahogany table-top (UK)	RS07PT2	Laboratory contaminant (Portugal)
JW07JP2	Metal surface of an armrest on a 1970's train (Japan)	RS07PT3	Laboratory contaminant (Portugal)
JW07JP4	Silicon floor-seal on a 1970's train (Japan)	RS07US5	Soil (North America)
JW07JP8	Silk toy hung on exterior of a building (Japan)	RS07US10	Soil (North America)
JW07JP13	Insect faeces on dead bamboo (Japan)		

a Strains were isolated on glycerol-supplemented and sucrose-supplemented MYPiA medium; see *Experimental procedures*. Strains RS07PT1, RS07PT2, RS07PT3, RS07US5 and RS07US10 were isolated by Ricardo dos Santos, Laboratório de Análises of the Instituto Superior Técnico, Portugal. Entries in bold correspond to strains selected for more detailed study (see Figs 1C–K, 2 and 3A).

b The third and fourth characters of strain designations indicate the year that sampling and isolation were carried out (i.e. 2005, 2006 or 2007).

For all environmental isolates and the named xerophile species (157 strains in total; see Tables 1 and 2) rates of hyphal extension were determined on low water-activity media containing one of a range of chemically diverse but biologically relevant solutes (see Fig. 1A and B). Generally strains from low-solute substrates grew down to similar water activities, and at comparable growth rates, to those from high-solute environments (data not shown). The solute that facilitated the optimum growth-rate varied depending on the fungal strain, but sucrose was most permissive for the majority of strains (Fig. 1A). By contrast, glycerol facilitated growth down to the lowest water-activity for more than 75% of strains (Fig. 1B) so we used glycerol-supplemented media to test the hypothesis that the stress parameter water activity does not always limit life on low water-activity substrates.

**Fig. 1:**
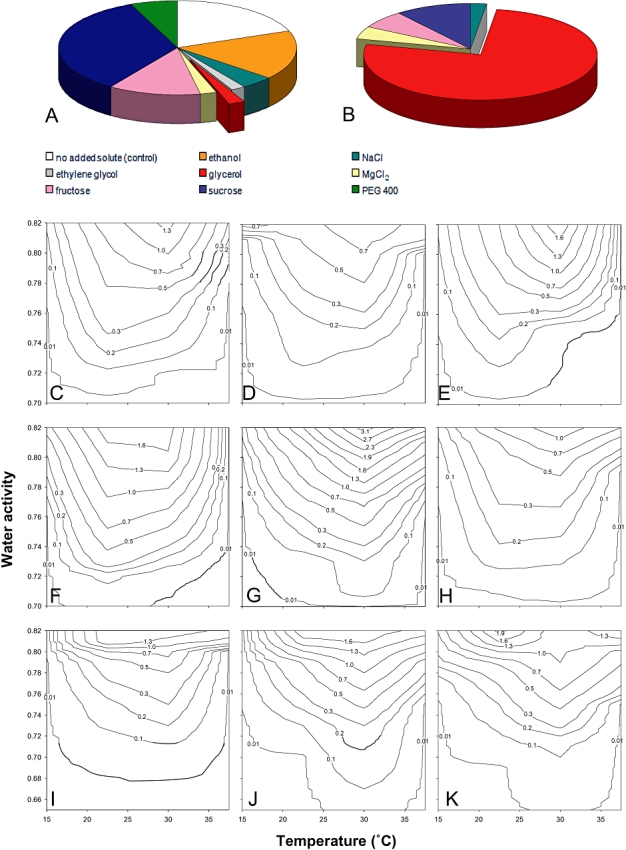
Stress tolerance of xerophilic fungi to (A and B) single stressors and (C–I) temperature : water-activity regimes. Proportion of the 157 fungal strains tested (see Tables 1 and 2) that (A) grew optimally and (B) grew to their water-activity minimum on media containing either no added solute (control) or those supplemented with ethanol, NaCl, ethylene glycol, glycerol, MgCl_2_, fructose, sucrose or PEG 400. For each medium type, fungi were grown over a range of concentrations from zero (control media) to the concentration limit that prevented growth (data not shown). For three fungal strains growth-rate data obtained from single-stressor screens were plotted according to the chaotropic or kosmotropic activity of media (see later). For C–K: growth profiles for the nine most xerophilic fungi incubated at 15, 20, 25, 30 and 37°C on glycerol-supplemented media [water-activity values ranged from 0.810 to 0.653; isopleth contours indicate growth rates (mm day^−1^)] and were plotted using Sigmaplot, Version 8.0. The fungal strains were (C) JH06THH, (D) JH06GBM, (E) JH06GBO, (F) JH06JPD, (G) *Aspergillus penicillioides* FRR 2179, (H) *Eurotium amstelodami* FRR 2792, (I) *Xeromyces bisporus* FRR 0025, (J) *X. bisporus* FRR 3443 and (K) *X. bisporus* FRR 2347 (see Tables 1 and 2).

Nine out of the 157 strains grew at ≤ 0.75 a_w_, and these had been isolated either from low-solute surfaces during the current study (strains JH06THH; JH06GBM; JH06GBO; JH06JPD from wooden surfaces, see Table 1) or from high-solute substrates by other research groups (strains *Aspergillus penicillioides* FRR 2179; *Eurotium amstelodami* FRR 2792; and three strains of *Xeromyces bisporus*: FRR 0025; FRR 3443; FRR 2347, see Table 2). Mycelial growth rates of these strains were quantified on glycerol-supplemented media over a matrix of temperature and water-activity values (Fig. 1C–K) in order to determine the limits of their biotic windows, and to obtain two-dimensional profiles of their growth phenotypes. We then determined the pH required for optimum growth over a range of water-activity values on glycerol-supplemented media in order to avoid inadvertently causing pH limitation. There were clear phenotypic differences between strains, but growth at low water activity was generally optimal at 30°C (see Fig. 1C–K) and pH 5.75 (data not shown) so these conditions were used throughout the study. Although hyphal growth has previously been recorded at ≤ 0.710 a_w_ (see Pitt and Christian, 1968; see later), only two strains grew on glycerol media at water-activity values significantly below 0.714 a_w_, regardless of temperature or pH (see Fig. 1J and K). The glycerol concentrations used in these media (i.e. ≤ 7.16 M) are consistent with the intra and/or extracellular concentrations to which microbial cells can be exposed in nature (Brown, 1990; Hallsworth and Magan, 1994a; de Jong *et al.*, 1997; Hallsworth, 1998; Zhuge *et al.*, 2001; Bardavid *et al.*, 2008). However, our data as well as earlier studies suggest that glycerol has inhibitory activities at molar concentrations (see Fig. 1J and K; Borowitz and Brown, 1974; Hallsworth *et al.*, 2007), and may act as a chaotropic stressor due to its unusual interactions with water and destabilizing effects on macromolecular structures (Borowitz and Brown, 1974; Hallsworth *et al.*, 2007; Chen *et al.*, 2009; F.D.L. Alves and J.E. Hallsworth, unpublished). We therefore formulated the hypothesis that solute activities other than water activity can determine the limits of microbial-cell function.

### Water activity did not limit life at low water activity

**Fig. 2:**
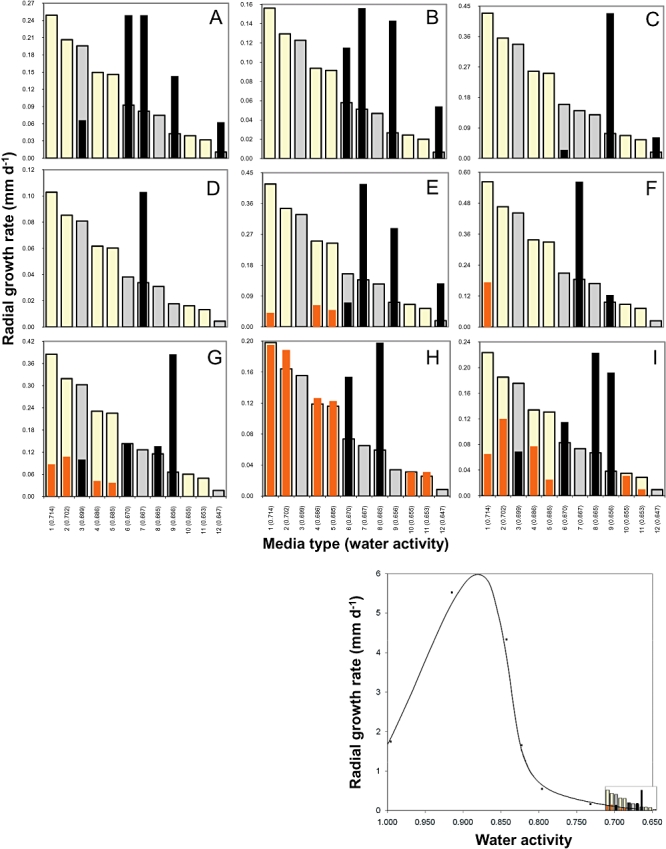
Growth rates at 30°C (A–I) for the nine selected xerophiles (see also Fig. 1C–K) on highly chaotropic (solid-orange columns) or neutral media (black columns) over a range of water-activity values (0.714–0.647; see also Table 3): Medium (1) glycerol (6.84 M), Medium (2) glycerol (7.06 M), Medium (3) glycerol (5.43 M), sucrose (0.73 M) plus NaNO_3_ (0.24 M), Medium (4) glycerol (7.48 M, and concentrations of malt extract, yeast extract and K_2_HPO_4_ that were 10-fold more dilute than those of the control medium), Medium (5) glycerol (7.48 M), Medium (6) glycerol (5.97 M), sucrose (0.73 M), Medium (7) glycerol (3.91 M), sucrose (0.73 M), NaCl (1.20 M) plus KCl (0.13 M), Medium (8) glucose (1.11 M), glycerol (4.34 M) plus fructose (1.11 M), Medium (9) glycerol (4.67 M), sucrose (0.73 M), NaCl (1.20 M) plus KCl (0.13 M), Medium (10) glycerol (7.60 M, pH 4), Medium (11) glycerol (7.60 M), and Medium (12) glycerol (6.19 M), NaCl (1.20 M) plus KCl (0.13 M). The pH of all media was 5.75 unless otherwise stated; chaotropic-activity values are shown in Table 3; the experiment was conducted on three independent occasions and variation of growth-rate values was within ±0.2 mm day^−1^. Growth rates are shown for the following fungal strains: (A) JH06THH, (B) JH06GBM, (C) JH06GBO, (D) JH06JPD, (E) *Aspergillus penicillioides* FRR 2179, (F) *Eurotium amstelodami* FRR 2792, (G) *Xeromyces bisporus* FRR 0025, (H) *X. bisporus* FRR 3443 and (I) *X. bisporus* FRR 2347 (see also Fig. 1C–K; Tables 1 and 2). Theoretical growth-rate values that were predicted based on the assumption that growth rates are proportional to medium water activity are shown for highly chaotropic media as shaded yellow columns and for neutral media as shaded grey columns. J. Growth curve of *X. bisporus* FRR 0025 over a full range of water activity values, showing the position of G (inset, lower right) in the context of the entire biotic window of this strain on glycerol-supplemented media at 30°C (see also Fig. 1I).

To test this hypothesis, we designed a range of 14 low water-activity media that were supplemented with either a chaotropic solute (fructose or glycerol) or combinations of glycerol and a number of other solutes: fructose and/or the kosmotropes sucrose, glucose, NaCl and KCl (Collins, 1997; Galinski *et al.*, 1997; see Table 3 and Fig. 2A–I). A number of recent studies provide evidence that some ions can penetrate the hydrophobic domains of macromolecular systems by shedding their hydration water and that, via their physical bulk, they disorder the tertiary/quaternary structure (see Sachs and Woolf, 2003), i.e. that – by our earlier definition – they act chaotropically. However, NaCl and KCl that were used to depress water activity in Media 7, 9 and 12 are kosmotropic (i.e. solutions of their ions have a net kosmotropic activity; see Table 3), and this is consistent with their stabilizing effects on membranes, proteins and other cellular structures (see Brown, 1990). The range of water-activity values tested (0.760–0.644 a_w_) lies at the extreme edge of the water-activity window for all nine xerophile strains under study, and growth optima at 30°C lay between 0.95 and 0.85, as shown for one *X. bisporus* strain in Fig. 2J. Remarkably there was no correlation between rates of radial extension for these nine fungi (the most xerophilic microbes thus far identified) on glycerol-containing media at ≤ 0.72 a_w_, and the water activity of their culture media (Fig. 2A–I). Generally, on glycerol-supplemented media at ≤ 0.85 a_w_, the growth rates of all strains decreased in proportion to medium water activity (e.g. see Fig. 2J). On the glycerol-supplemented medium at 0.644 a_w_ and the fructose-supplemented medium at 0.760 a_w_ (despite the relatively high water activity of the latter; see Table 3) there was no hyphal growth of any xerophile strain (therefore data are not shown for Media 13 or 14 in Fig. 2). Paradoxically, for Medium 1 (at 0.714 a_w_) eight out of the nine fungal strains either failed to grow (Fig. 2A–D) or grew 65–90% more slowly than predicted (see Fig. 2E–G and I), whereas at lower water-activities (0.670–0.647) growth rates were up to 580% greater than predicted (i.e. the mixed-solute Media 6–9 and 12; see Fig. 2A–I).

**Table 3:** Chaotropic-activity and water-activity values for solutes and solute combinations used to supplement growth media.a

	Added solute(s); concentration [M]							
Medium designation^a^	Glycerol	NaCl	KCl	Fructose	Glucose	Sucrose	Chaotropic activity (kJ kg^–1^)^b^	Water activity^c^
1	6.84	0	0	0	0	0	Highly chaotropic (15.27)	0.714
2	7.06	0	0	0	0	0	Highly chaotropic (16.64)	0.702
3	5.34	0	0	0	0	0.73	Relatively neutral (12.48^d^)	0.699
4	7.48	0	0	0	0	0	Highly chaotropic (18.05)	0.686
5	7.48	0	0	0	0	0	Highly chaotropic (18.05)	0.685
6	5.97	0	0	0	0	0.73	Relatively neutral (11.11^d^)	0.670
7	3.91	1.20	0.13	0	0	0.73	Relatively neutral (−2.75d,e)	0.667
8	4.34	0	0	1.11	1.11	0	Relatively neutral (9.73^d^)	0.665
9	4.67	1.20	0.13	0	0	0.73	Relatively neutral (−2.75d,e)	0.656
10	7.60	0	0	0	0	0	Highly chaotropic (28.80)	0.655
11	7.60	0	0	0	0	0	Highly chaotropic (20.80)	0.653
12	6.19	1.20	0.13	0	0	0	Relatively neutral (2.79^d^)	0.647
13	7.65	0	0	0	0	0	Highly chaotropic (20.88)	0.644
14	0	0	0	4.80	0	0	Highly chaotropic (20.80)	0.760

a See Fig. 2A–I. The pH of all media was 5.75, except for Medium 4 (pH 4).

b See Hallsworth and colleagues (2003a).

c Measured at 30°C.

d Extrapolated from agar gel-point curve.

e Media were slightly kosmotropic so the activity value is negative.

The lowest water activity previously reported for sustained growth of fungi was 0.656: for *X. bisporus* after a 90 day incubation period (Pitt and Christian, 1968). By comparison several fungal strains grew in the current study at 0.656 a_w_, and hyphal growth was observed at this water activity for one strain after only 11 days (Fig. 2F; Table 4). Remarkably we observed growth at water-activity values as low as 0.647, and did so in as little as 5–8 weeks, on mixed-solute media (see Fig. 2A–I; Table 4). Furthermore, four out of the five strains that were able to grow at 0.647 a_w_ had been isolated from low-solute surfaces (in the current study; see Table 1) and were therefore more xerophilic than all but one of the strains isolated from high-solute environments during the past 100 years (see Fig. 2A–I; Table 4). One fungal strain, isolated in 2006 from the wooden (sycamore) surface of a 19th Century, kitchen chopping-block (JH06NIM; see Table 1), was observed to be growing at 0.647 a_w_ after only 34 days incubation at 30°C (Fig. 2B; Table 4).

**Table 4:** Fungal strains capable of hyphal growth ≤ 0.71 water activity.^a^

Species and/or strain designation	Nature of substrate of origin^b^	Lowest recorded water activity for hyphal growth^c^	Earliest observation of hyphal growth (day)	Rate of hyphal extension (mm day^−1^)	Method used to reduce water activity (reference^d^)	Chaotropic or kosmotropic activity of culture medium (kJ kg^−1^)
JH06GBM	L-S	0.647	34	0.05	Glycerol (6.19 M), NaCl (1.20 M), KCl (0.13 M)^e^	Relatively neutral (2.79)
*Aspergillus penicillioides* FRR 2179	H-S	0.647	46	0.13	Glycerol (6.19 M), NaCl (1.20 M), KCl (0.13 M)^e^	Relatively neutral (2.79)
JH06THH	L-S	0.647	46	0.06	Glycerol (6.19 M), NaCl (1.20 M), KCl (0.13 M)^e^	Relatively neutral (2.79)
JH06GBO	L-S	0.647	60	0.06	Glycerol (6.19 M), NaCl (1.20 M), KCl (0.13 M)^e^	Relatively neutral (2.79)
JH06THJ	L-S	0.647	60	0.03	Glycerol (6.19 M), NaCl (1.20 M), KCl (0.13 M)^e^	Relatively neutral (2.79)
*Xeromyces bisporus* FRR 3443	H-S	0.653	41	0.03	Glycerol (7.60 M)^e^	Highly chaotropic (20.80)
*X. bisporus* FRR 2347	H-S	0.653	41	0.01	Glycerol (7.60 M)^e^	Highly chaotropic (20.80)
*X. bisporus* FRR 1522	H-S	0.653	41	0.01	Glycerol (7.60 M)^e^	Highly chaotropic (20.80)
*Eurotium amstelodami* FRR 2792	H-S	0.656	11	0.12	Glycerol (4.67 M), sucrose (0.73 M), NaCl (1.20 M), KCl (0.13 M)^e^	Relatively neutral (−2.75)^f^
*X. bisporus* FRR 0025	H-S	0.656	22	0.39	Glycerol (4.67 M), sucrose (0.73 M), NaCl (1.20 M), KCl (0.13 M)^e^	Relatively neutral (−2.75)^f^
*A. penicillioides* FRR 3722	H-S	0.656	29	0.13	Glycerol (4.67 M), sucrose (0.73 M), NaCl (1.20 M), KCl (0.13 M)^e^	Relatively neutral (−2.75)^f^
*E. amstelodami* FRR 0475	H-S	0.656	29	0.12	Glycerol (4.67 M), sucrose (0.73 M), NaCl (1.20 M), KCl (0.13 M)^e^	Relatively neutral (−2.75)^f^
JH06THI	L-S	0.656	60	0.03	Glycerol (4.67 M), sucrose (0.73 M), NaCl (1.20 M), KCl (0.13 M)^e^	Relatively neutral (−2.75)^f^
*X. bisporus*	H-S	0.656	90	Not quantified	Thin layer of medium on a glass surface in a humidity-controlled chamber (Pitt and Christian, 1968)^g^	Not quantified
*X. bisporus*	H-S	0.663	120	Not quantified	Thin layer of medium on a glass surface in a humidity-controlled chamber (Pitt and Christian, 1968)^g^	Not quantified
JH06JPD	L-S	0.667	11	0.10	Glycerol (3.91 M), sucrose (0.73 M), NaCl (1.20 M), KCl (0.13 M)^e^	Relatively neutral (−2.75)^f^
JH07JP128	L-S	0.667	94	0.02	Glycerol (3.91 M), sucrose (0.73 M), NaCl (1.20 M), KCl (0.13 M)^e^	Relatively neutral (−2.75)^f^
*Eurotium halophilicum* FRR 2471	H-S	0.675	38	Not quantified	Equal weights of glucose and fructose added to growth media (Andrews and Pitt, 1987)^h^	Not quantified
*Chrysosporium xerophilium* FRR 0530	H-S	0.686	118	0.01	Glycerol (7.48 M)^e^	Highly chaotropic (18.05)
*Chrysosporium fastidium*	H-S	0.697	64	Not quantified	Thin layer of medium on a glass surface in a humidity-controlled chamber (Pitt and Christian, 1968)^g^	Not quantified
*C. xerophilium*	H-S	0.708	80	Not quantified	Thin layer of medium on a glass surface in a humidity-controlled chamber (Pitt and Christian, 1968)^g^	Not quantified
*Eurotium chevalieri* PIL 119	H-S	0.710	16	0.1	A thin layer of medium enclosed in a humidity-controlled, bung-sealed glass test tube (Ayerst, 1969)^i^	Not quantified
*E. amstelodami* PIL 120	H-S	0.710	32	0.1	A thin layer of medium enclosed in a humidity-controlled, bung-sealed glass test tube (Ayerst, 1969)^j^	Not quantified

a Data for the yellow-shaded entries were obtained from the current study.

b H-S = isolated from a high-solute substrate; L-S = isolated from a low-solute surface.

c Compiled using data from the current study and from published xerophile studies; refs. Pitt and Christian (1968); Ayerst (1969); Andrews and Pitt (1987).

d Data were obtained from the current study unless otherwise stated.

e The culture medium was MYPiA (pH 5.75, 30°C); see *Experimental procedures*.

f N.B. Media were slightly kosmotropic so the activity value is negative.

g The culture medium was Czapek Invert Malic Agar (pH 3.8, 25°C).

h The culture medium was Yeast Nitrogen Base + 2% glucose w/v + 2% agar w/v.

i The culture medium was Malt Extract Agar; MEA (30–40°C).

j The culture medium was MEA (24–30°C).

For a given fungal species, the lower water-activity limit for the germination of propagules is typically lower than that for hyphal growth (Pitt, 1975). However, numerous studies of spore germination of xerophiles at low water-activities (see Table S1) have found that growth ceases upon germ-tube production (Pitt and Christian, 1968). Whereas a number reviews cite germ-tube formation by *X. bisporus* spores at 0.605 a_w_ as evidence of cellular function at ultra-low water activity (Pitt, 1975; Grant, 2004; see also Table S1), further hyphal growth and mycelium development were not recorded (Pitt and Christian, 1968). In the current study hyphal growth of *A. penicillioides* and *E. amstelodami* occurred at considerably lower water-activity values (0.647 and 0.656 a_w_ on mixed-solute media; see Fig. 2E and F; Table 4) than those previously reported for germination (i.e. 0.680 and 0.703 respectively; see Table S1). For each xerophile strain at water-activity values below their growth optimum, growth rates were proportionally reduced (for an example, see Fig. 2J). However, at extremely low water activity (≤ 0.72 a_w_), growth rates were no longer proportional to water activity so we concluded that other stress parameters limited cellular activity. Furthermore, we asked the scientific questions whether the chaotropicity of glycerol-supplemented media (6.84–7.65 M glycerol) limited hyphal growth at low water activity, and whether this inhibition was reversed by the kosmotropic activity of other substances present in the mixed-solute media (3.91–6.19 M glycerol).

### Chaotropic compounds limited cell function, but their effects were reversible

**Fig. 3:**
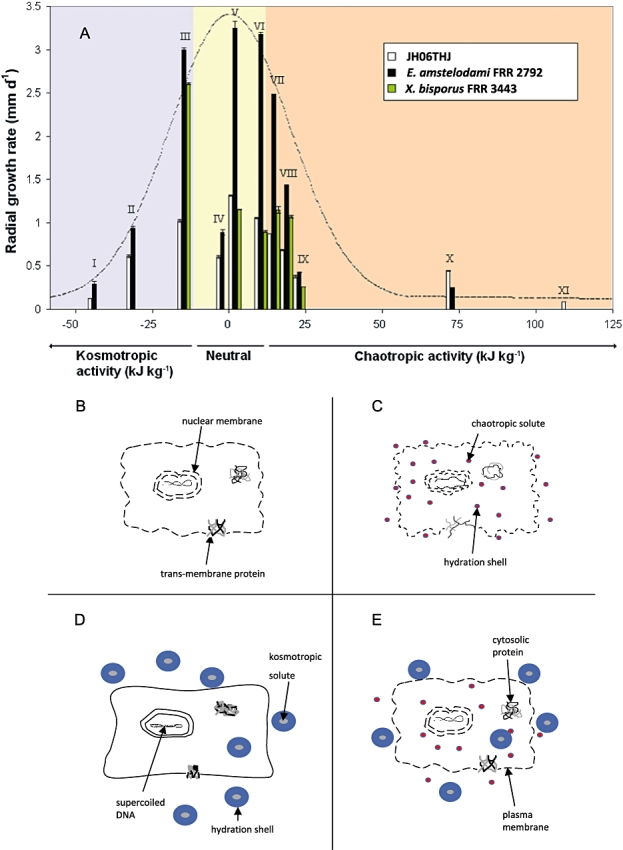
Growth rates of three representative xerophilic fungi (A); *Xeromyces bisporus* FRR 3443, *Eurotium amstelodami* FRR 2792, and isolate JH06THAJ (see Tables 1 and 2) in relation to chaotropic and kosmotropic activities of culture media: (I) NaCl (4.28 M, 0.775 a_w_), (II) NaCl (3.59 M, 0.812 a_w_), (III) sucrose (2.34 M, 0.831 a_w_), (IV) PEG 400 (1.25 M, 0.855 a_w_), (V) glycerol (4.90 M, 0.828 a_w_), (VI) fructose (3.51 M, 0.829 a_w_), (VII) fructose (3.94 M, 0.804 a_w_), (VIII) fructose (4.36 M, 0.791 a_w_), (IX) glycerol (6.66 M, 0.747 a_w_), (X) ammonium nitrate (4.30 M, 0.855 a_w_) and (XI) ammonium nitrate (5.15 M, 0.817 a_w_). Values are means of three replicates and bars represent standard errors. The data approximate to a Normal distribution (see dotted line), although it may be that the osmotic stress or other stress parameters associated with kosmotropic stressors ultimately limit hyphal growth. Diagrammatic illustrations (B–E) of the way in which chaotropic and kosmotropic activities impact on macromolecule and membrane structure in relation to an unstressed cell (B); in a chaotrope (e.g. urea)-stressed cell (C), a kosmotrope (e.g. sucrose)-stressed cell (D), and a cell exposed to both chaotropes and kosmotropes (E).

The fructose-supplemented medium and the seven glycerol-supplemented media were found to have chaotropic-activity values of 15–21 kJ kg solution^−1^ (Table 3); values that are consistent with the chaotropicity limits for other microbial species (see Hallsworth *et al.*, 2007). However, the activity values of the six mixed-solute media that contained kosmotropes were relatively neutral (12.48 to −2.75 kJ kg solution^−1^; see Table 3). Generally there were either low rates of radial extension on chaotropic media, or no growth at all (for glycerol-only media see Fig. 2A–I, orange columns; for fructose-only media data not shown). By contrast, remarkably high growth-rate values were obtained on the other media that were neutral or mildly chaotropic, and these were several hundred per cent higher than those predicted from water-activity values (see Fig. 2A–I, black columns). There was a strong inverse correlation between chaotropic activity and fungal growth (see Figs 2A–I and 3A): three strains that were able to grow down to 0.647 a_w_ did not grow on any glycerol-supplemented media even at the relatively less-stressful water activity of 0.714 (Fig. 2A–C).

Glycerol, which is neutral or only weakly chaotropic below concentrations of 3–4 M (F.D.L. Alves and J.E. Hallsworth, unpublished), is widely known for its activities as a stress protectant that can both protect the structure and function of cellular macromolecules, and act as an intracellular osmolyte to control cell turgor (Brown, 1990; Dashnau *et al.*, 2006). At higher concentrations (≥ 6 M), however, we have demonstrated the extreme chaotropicity of glycerol (Table 3). On high-solute substrates xerophile cells can fail due to the prohibitive energy expenditure required to retain the intracellular glycerol that is needed as an osmolyte (Hocking, 1993). We propose that glycerol itself can disorder and permeabilize the plasma membrane, via its chaotropic activity, thereby resulting in the leakage of this protectant from the cell.

Whereas the biochemical mechanisms by which chaotropic solutes disorder cellular macromolecules are not yet fully understood (see above), we have illustrated the structural consequences for macromolecular systems in a cell stressed by a chaotropic solute, and the way in which kosmotropic solutes counter chaotrope-induced stress (Fig. 3B–E). For a cell growing under optimal conditions macromolecular structures and activities will presumably be optimal (see Fig. 3B). We propose that a glycerol-stressed cell, like those on Media 1, 2, 4, 5, 10, 11 in Fig. 2A–I, has disordered macromolecular and membrane structures, and increased membrane permeability (see Fig. 3C). Conversely, a kosmotrope-stressed cell, such as those on high-sucrose media, is likely to have highly ordered macromolecular structures (e.g. a rigidified plasma membrane), reduced membrane permeability, and may also be osmotically stressed (see Fig. 3D). We propose that a cell simultaneously exposed to opposing chaotropic and kosmotropic activities (such that the net effect is close to neutral; Media 3, 6, 7, 8, 9, 12 in Fig. 2A–I; Table 3), has macromolecular structures that are neither highly disordered nor highly ordered (Fig. 3E). Superficially, the appearance of macromolecular structures in an optimally growing cell and a stressed cell exposed to a chaotrope-kosmotrope mixture (Media 3, 6, 7, 8, 9, 12 in Fig. 2A–I) are qualitatively similar (Fig. 3B and D). However, the metabolic activity and growth rate of the latter can be orders of magnitude lower (see Fig. 2J) than that of the optimally growing cell illustrated in Fig. 3B, because it is subjected to the inhibitory effects of solutes such as lowered intra/extracellular water activity and/or solute-crowding effects, and furthermore the opposing chaotropic/kosmotropic solute activities may not be evenly balanced.

In summary, the data presented here (Figs 1, 2 and 3A; Table 3) support the hypothesis that the chaotropic activity of glycerol, not the stress parameter water activity, limits cell metabolism for these xerophilic fungi at ≤ 0.72 a_w_. This is consistent with evidence from other reports that chaotropicity limits microbial function (Hallsworth, 1998; 2003a; Duda *et al.*, 2004; Lo Nostro *et al.*, 2005), with a study showing that compatible solutes can reduce ethanol stress in conidia of *Aspergillus nidulans* (Hallsworth *et al.*, 2003b), and with our recent studies of halophilic prokaryotes which demonstrated that the macromolecule-structuring activities of kosmotropic salts can reduce or reverse the inhibitory effects of chaotropic salts (Hallsworth *et al.*, 2007).

### *Chaophiles*: a new class of stress-tolerant organism

There are several classes of solute-tolerant microbe: salt-tolerant halophiles, sugar-tolerant osmophiles, and xerophiles that tolerate low water activity (whereas conceptually distinct these ecophysiological groupings may not be mutually exclusive; see Brown, 1990). Although we recently proposed a new ecophysiological grouping, *chaophilic* microbes, a search for chaotrope-tolerant strains in samples taken from the hypersaline deep-sea Discovery Basin proved fruitless (Hallsworth *et al.*, 2007). The current study provides the first evidence, to our knowledge, for an apparent chaotropicity preference of physiologically active cells (see Fig. 2G–I; Table 4). Four strains failed to grow on any chaotropic media (Fig. 2A–D), however, strains of *X. bisporus* were able to tolerate all highly chaotropic media (up to 7.60 M glycerol; Fig. 2G–I). For example, *X. bisporus* strain FRR 3443 grew fastest under chaotropic conditions (on Media 1 and 2), even grew at 0.653 a_w_ on a chaotropic glycerol-supplemented medium (Medium 11), and failed to grow on the three mixed-solute media that were either very weakly chaotropic or slightly kosmotropic (Media 3, 7 and 9, see Fig. 2H; Table 3). Whereas this strain also grew on Media 6 and 8 (Fig. 2H) that were relatively neutral, it is noteworthy that these two media were more chaotropic (11.11 and 9.73 kJ kg^−1^ respectively) than the majority of the other media allocated to the neutral or slightly kosmotropic categories (Table 3). High temperatures, like chaotropic substances, disorder cellular membranes and other macromolecular structures (Hallsworth *et al.*, 2003a), and growth of *X. bisporus* strains on high-glycerol media was optimal at 30°C (Fig. 1I–K), but at 22°C for the other xerophile strains (Fig. 1C-H). Although *X. bisporus* strains were apparently more xerophilic at higher temperature, this phenotype may actually indicate a preference for conditions that disorder macromolecular and membrane structures. Collectively, these data provide evidence for a new class of extremophilic microbes that are *chaotolerant* or *chaophilic*.

### Implications and conclusions

**Fig. 4:**
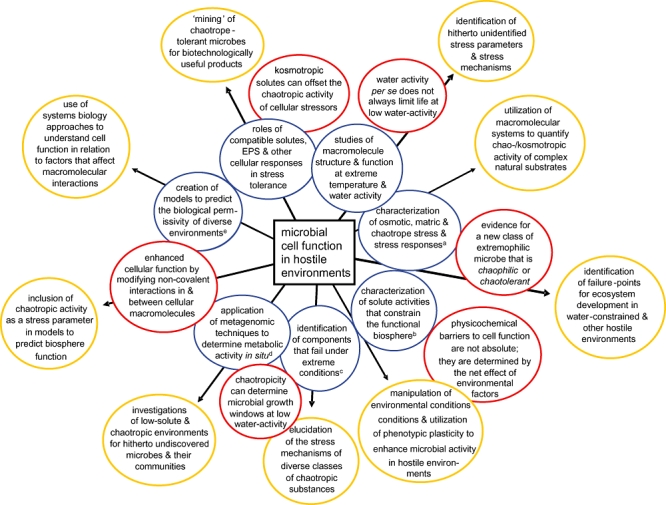
Representation of scientific progress towards understanding the limits of microbial function in hostile environments in relation to earlier studies (blue), the current study (red), and further studies that are needed (yellow); a. Dutrochet (1826); Griffin (1977); Hallsworth and colleagues (2003a); b. Scott (1957); Hallsworth and colleagues (2007); c. Hocking (1993); Ferrer and colleagues (2003); d. e.g. Hallsworth and colleagues (2007); and e. Marion and Kargel (2008).

We already have an understanding of environmentally relevant solute stresses [osmotic stress (Dutrochet, 1826), matric stress (Griffin, 1977) and chaotrope-induced water stress (Hallsworth *et al.*, 2003a)]; how chaotropic agents determine the limits of macromolecule function (see Hallsworth *et al.*, 2003a; 2007; Duda *et al.*, 2004); indications of the cellular components that fail under extreme forms of stress (see current study; Hocking, 1993; Ferrer *et al.*, 2003); and other factors that determine the limits of microbial function in hostile environments (see Fig. 4; Pitt, 1975; Golyshina *et al.*, 2006; Hallsworth *et al.*, 2007; Marris, 2008). The current study illustrates how hitherto unidentified stress parameters can limit microbial cell function under certain environmental conditions, and may thereby constrain the biosphere in specific locations. Further work is needed to identify and characterize the stress mechanisms that act as failure points for ecosystems in hostile environments (Fig. 4). Many informative studies of the geochemical composition of extreme environments have already been carried out, including those of other planets (Mustard *et al.*, 2008). Although chaotropicity has been shown to limit the functional biosphere in specific locations on Earth (Hallsworth *et al.*, 2007), this stress parameter has not yet been factored into the mathematical models used to predict the feasibility of life in as-yet-unexplored environments on Earth or other planetary bodies (Beaty and Buxbaum, 2006; Marion and Kargel, 2008; Mustard *et al.*, 2008; Tosca *et al.*, 2008). We believe that chaotropicity should be accounted for in future models that aim to predict what types of environment can potentially support cellular activity (Fig. 4).

Global climate change, and changes in land-use, have accelerated the expansion of biologically hostile arid and semiarid regions over the past 30 years (Thomas *et al.*, 2005; Seager *et al.*, 2007). Polluted environments also represent a challenge to microbes that are exposed to the chaotropic activities of xenobiotics (Hallsworth *et al.*, 2003a). Microbes in other natural habitats, as well as substrates used in industrial processes, may also be subjected to low water-activity conditions and/or high concentrations of chaotropic stressors such as formamide, ethanol, urea, ethylene glycol, butanol, NH_4_NO_3_, glycerol, phenol, MgCl_2_, CaCl_2_ and sodium benzoate (Brown, 1990; Hallsworth, 1998; 2003a; 2007; Bardavid *et al.*, 2008). For both low water-activity and highly chaotropic substrates, analysis of the ways in which these (and other) stress parameters interact to limit biological activity can shed light on how to optimize or eliminate microbial activity, as required. Chaotropes and high temperatures disorder cellular structures, whereas kosmotropes and low temperatures have a stabilizing/ordering effect (Hamaguchi and Geiduschek, 1962; Collins, 1997; Hallsworth *et al.*, 2003a) and we utilized the counteracting solute activities of glycerol and kosmotropic substances in order to extend the biotic window for xerophile growth at low water activity (Fig. 2A–I; Table 4). It may be that microbial cells in laboratory culture could, and that cells in nature do, function below the water-activity limit of 0.647 established in the present study (Fig. 2), and that we have not yet understood how to manipulate solute activities sufficiently well to maintain macromolecular function under high-solute conditions. Furthermore, the vast majority of microbes cannot be cultivated *in vitro* (Whitman *et al.*, 1998; Rutz and Kieft, 2004; Ward and Fraser, 2005) and some of these may remain elusive as long as the physicochemical parameters that determine their biotic windows for growth are poorly understood. We propose that manipulation of solute activities will facilitate the cultivation and study of numerous microbial species that can currently only be detected *in situ* (using metagenomic techniques).

Knowledge-based approaches to manipulating environmental conditions could lead to strategies for the regeneration of desertified regions (see Kashangura *et al.*, 2006), and for providing both food and biofuels to support human population whilst maintaining a sustainable biosphere. Diverse approaches based on manipulation of environmental conditions or stress parameters have already given rise to quantum improvements in the growth windows of mesophilic species (see Hallsworth and Magan, 1994b; 1995; Thomas *et al.*, 1994; Hallsworth *et al.*, 2007); there is evidence that kosmotropic substances reduce ethanol stress in yeast (see Hallsworth, 1998), and that kosmotropic ions can increase *Halobacterium* activity under chaotropic conditions (see Oren, 1983; Hallsworth *et al.*, 2007). The bioremediation of chaotrope-polluted soils (Hallsworth *et al.*, 2003a) may be most efficient at temperatures low enough to minimize the chaotrope-induced disordering of cellular structures. The effectiveness and efficiency of products and processes such as biocides, food preservatives (e.g. sodium benzoate), food and drinks fermentations, bioalcohol production from microbes (Hallsworth, 1998), and industrial biocatalysis in solvent systems, which utilize or generate chaotropic solutes could be enhanced via manipulation of solute activities. Further studies are needed so that more effective interventions can be made based on exploitation of phenotypic plasticity, employing recombinant technologies and/or systems biology approaches to obtaining stress-resistant cells (see Fig. 4).

Xerophilic fungi have most commonly been isolated from kosmotrope-containing substrates (Pitt, 1975), so chaophilic microbes in nature may have thus far gone unnoticed. Further, high-solute habitats have typically been the focus of searches for xerophilic microbes so countless xerophilic species (and indeed whole communities) on low-solute surfaces may have been overlooked. It is intriguing to speculate what proportion of xerophilic fungi in nature are also chaophilic, and what proportion are restricted to either high-solute or low-solute substrates. Furthermore, extremophilic fungi already act as important biotechnological resource (Archer and Peberdy, 1997), and chaotolerant species and/or their enzymes may have potential for diverse applications. One focus of our ongoing studies is to identify novel stress parameters that prevent life processes: it may be possible to further enhance microbial function, and ecosystem development, in hostile environments once the stress biology of microbes has been more completely elucidated.

## Experimental procedures

### Sampling strategies and environmental isolates

Fungi were isolated from diverse environments (see Table 1) using sterile cotton-tip swabs and inoculated onto slants of Malt-Extract, Yeast-Extract Phosphate Agar [MYPiA; 1% Malt-Extract w/v (Oxoid, UK), 1% Yeast-Extract w/v (Oxoid, UK), 1.5% Agar w/v (Acros, USA), 0.1% K_2_HPO_4_ w/v] supplemented with 5 M glycerol (0.845 a_w_) in 1.8 ml, internal-thread cryovials and transferred, once back in the laboratory, to Petri plates containing MYPiA supplemented with glycerol (5 M; 0.845 a_w_) or sucrose (2.2 M; 0.884 a_w_). Petri plates were incubated at temperatures between 20°C and 30°C in sealed bags (see below) and checked periodically for up to 6 months. Upon visual inspection, all isolates that had grown were subcultured onto MYPiA supplemented with 6.52 M glycerol, and incubated at 30°C.

### Named xerophile species

Named xerophile species were obtained from Culture Collection of Yeasts (CCY, Bratislava, Slovakia), German Collection of Microorganisms and Cell Cultures (DSMZ, Braunschweig, Germany), Extremophilic Fungi Culture Collection (EXF, Ljubljana, Slovakia), Food Research Ryde (FRR, North Ryde, Australia), International Mycological Institute (IMI, Egham, UK), and University of Western Ontario, UWOPS Collection (Ontario, Canada); see Table 2. Cultures were maintained on MYPiA supplemented with either 2.78 M glucose (0.911 a_w_) or 2.2 M sucrose (0.884 a_w_) in sealed bags (see below) and incubated at 30°C.

### Culture conditions and growth-rate determinations

Throughout this study, all media were sterilized in Schott bottles in a water bath (100°C, 60 min), cooled to within 7.5°C of the medium gel-point, and then poured into 9 cm vented Petri plates; inoculations were carried out using 4 mm diameter plug from the periphery of actively growing cultures growing on MYPiA media supplemented with 5.43 M glycerol; and plates containing identical media were sealed in polyethylene bags to maintain a constant water activity (see Hallsworth *et al.*, 2003b). Growth was assessed at periodic intervals by taking two measurements of colony diameter (in perpendicular directions), that were used to calculate rates of radial extension as described previously (Pitt and Hocking, 1977; Hallsworth and Magan, 1994a). Two-dimensional stress-tolerance profiles were plotted as described previously (Hallsworth and Magan, 1999) and mean values were plotted, and variation indicated in each display. Growth of yeast was carried out on the same media and quantified using spot tests as detailed by Albertyn and colleagues (1994).

### Modification of media to investigate stress tolerance

#### 

##### 

###### Single-stressor solute-tolerance screen

All environmental isolates and named fungal strains were screened for stress tolerance on MYPiA media supplemented with different solutes over a range of concentrations; either ethanol (0.4–1.3 M), NaCl (2.1–3.4 M), ethylene glycol (2.0–3.4 M), glycerol (2.5–5.0 M), MgCl_2_ (0.7–1.5 M), fructose (3.0–4.8 M), sucrose (1.1–2.1 M), or polyethylene glycol (PEG) 400 (0.8–1.3 M), without addition of pH buffer, and incubated at 30°C for a period of up to 90 days. All inoculations were carried out in triplicate.

###### pH-tolerance study

All environmental isolates and named fungal strains were grown on MYPiA media supplemented with glycerol (at 3.8 M; 0.92 a_w_ and 4.4 M; 0.88 a_w_ respectively) and buffered to pH values of: 3.75, 4.5, 5.75 (citric acid; Na_2_HPO_4_), 6, 6.75 (MES; NaOH) and 7.5 (Hepes; NaOH; see Hallsworth and Magan, 1996). The pH of each medium was adjusted prior to autoclaving using appropriate buffers then measured postautoclave using a *Mettler Toledo Seven Easy*, pH-probe (Switzerland).

###### Temperature: water-activity growth-response study

The nine most xerophilic strains were inoculated onto MYPiA media supplemented with four different concentrations of glycerol (5.43, 6.19, 6.84 and 7.44 M) with water activity values ranging from 0.81 and 0.65 (see Fig. 1C–K) and incubated at 15, 22.5, 30 and 37.5°C. Quantification of water activity is described below.

###### Mixed-solute media for limits-of-cell function at low water-activity study

The nine xerophilic strains were inoculated onto 14 ultra-low water-activity media (0.714–0.644 a_w_), consisting of MYPiA media supplemented with combinations of glycerol, fructose, or glycerol plus kosmotropic solutes (see Fig. 2, Table 3, Fig. S1). The pH of all media was adjusted to 5.75, unless stated otherwise, using citric acid: Na_2_HPO_4_ buffer; following inoculation Petri plates were incubated at 30°C. For water-activity and chaotropic-activity values of Media 1–14 see Table 3; the methodologies used to obtain these values are described below.

### Quantification of water activity

The water-activity values of media were measured at 30°C or, if different, at the temperature of incubation using a Novasina IC-II water-activity machine fitted with an alcohol-resistant humidity sensor and eVALC alcohol filter (Novasina, Pfäffikon, Switzerland), as described previously (Hallsworth and Nomura, 1999). This equipment was calibrated using saturated salt solutions of known water activity (Winston and Bates, 1960). Values were determined three times using replicate solutions made up on separate occasions. The variation of replicate values was within ±0.002 a_w_.

### Determination of chaotropic activity

The chaotropic activity of solute(s) used to supplement growth media (see Table 3) was measured as a function of their ability to destabilize the polysaccharide macromolecule agar (Extra-Pure Reagent-grade agar, gel strength 600–700 g cm^−2^, from Nacalai Tesque, Kyoto, Japan), and thereby lower gel-point (Hallsworth *et al.*, 2003a). Agar was melted in distilled water, cooled to 55°C, and added to a solution of the solute or solute-mixture to be tested, also at 55°C, to give a final concentration of agar of 1.5% w/v and concentration(s) of solute(s) as used for the growth study. The agar–compound solutions were allowed to cool gradually and the gel-point temperature (± 0.3°C) was recorded using a temperature probe (Jenway, UK). The gel points determined were used to calculate the chaotropic activity of each compound in kJ kg^−1^ (mole added compound)^−1^, based on the fact that the heat capacity for a 1.5% agar w/v gel is 4.15 kJ kg^−1^ °C^−1^ (see Hallsworth *et al.*, 2003a).

## Supporting information

**Fig. S1.** Flow-chart to illustrate the progression of research activities during the current study.

**Table S1.** Fungal species capable of germination ≤ 0.71 water activity.

## References

[b1] Albertyn J, Hohmann S, Thevelein JM, Prior BA (1994). Gpd1, which encodes glycerol-3-phosphate dehydrogenase, is essential for growth under osmotic-stress in *Saccharomyces-cerevisiae*, and its expression is regulated by the high-osmolarity glycerol response pathway. Mol Cell Biol.

[b2] Andrews S, Pitt JI (1987). Further-studies on the water relations of xerophilic fungi, including some halophiles. J Gen Microbiol.

[b3] Archer DB, Peberdy JF (1997). The molecular biology of secreted enzyme production by fungi. Crit Rev Biotechnol.

[b4] Austin AT, Yahdjian L, Stark JM, Belnap J, Porporato A, Norton U (2004). Water pulses and biogeochemical cycles in arid and semiarid ecosystems. Oecologia.

[b5] Ayerst G (1969). The effects of moisture and temperature on growth and spore germination in some fungi. J Stored Prod Res.

[b6] Ball P (2008). Water as an active constituent in cell biology. Chem Rev.

[b7] Bardavid RE, Khristo P, Oren A (2008). Interrelationships between *Dunaliella* and halophilic prokaryotes in saltern crystallizer ponds. Extremophiles.

[b8] Beaty DW, Buxbaum KL (2006). Findings of the Mars Special Regions Science Analysis Group. Astrobiology.

[b9] Boidin J, Pignal MC, Mermier F, Arpin M (1963). Quelques levures camerounaises. Cahiers la Maboké.

[b10] Borowitz LJ, Brown AD (1974). Salt relations of marine and halophilic species of unicellular green-alga, *Dunaliella*– role of glycerol as a compatible solute. Arch Microbiol.

[b11] Brown AD (1990). Microbial Water Stress Physiology – Principles and Perspectives.

[b12] Chaplin M (2006). Opinion – Do we underestimate the importance of water in cell biology?. Nat Rev Mol Cell Biol.

[b13] Chen C, Li WZ, Song YC, Yang J (2009). Hydrogen bonding analysis of glycerol aqueous solutions: a molecular dynamics simulation study. J Mol Liquids.

[b14] Collins KD (1997). Charge density-dependent strength of hydration and biological structure. Biophys J.

[b15] Collins SL, Sinsabaugh RL, Crenshaw C, Green L, Porras-Alfaro A, Stursova M, Zeglin LH (2008). Pulse dynamics and microbial processes in aridland ecosystems. J Ecol.

[b16] Corry JEL (1976). Sugar and polyol permeability of *Salmonella* and osmophilic yeast-cell membranes measured by turbidimetry, and its relation to heat-resistance. J Appl Bacteriol.

[b17] Dashnau JL, Nucci NV, Sharp KA, Vanderkooi JM (2006). Hydrogen bonding and the cryoprotective properties of glycerol. J Phys Chem B.

[b18] Dixit S, Crain J, Poon WCK, Finney JL, Soper AK (2002). Molecular segregation observed in a concentrated alcohol-water solution. Nature.

[b19] Duda VI, Danilevich VN, Suzina NE, Shorokhova AP, Dmitriev VV, Mokhova ON, Akimov VN (2004). Changes in the fine structure of microbial cells induced by chaotropic salts. Microbiology (Russia).

[b20] Dutrochet H (1826). L'Agent Immédiat Du Mouvement Vital Dévoilé Dans La Nature Et Dans Son Mode d'action, Chez Les Végétaux Et Les Animaux.

[b21] Fennell DI, Raper KB (1955). New species and varieties of *Aspergillus*. Mycologia.

[b22] Ferrer M, Chernikova TN, Yakimov MM, Golyshin PN, Timmis KN (2003). Chaperonins govern growth of *Escherichia coli* at low temperatures. Nat Biotechnol.

[b23] Galinski EA, Stein M, Amendt B, Kinder M (1997). The kosmotropic (structure-forming) effect of compensatory solutes. Comp Biochem Physiol a Physiol.

[b24] Gock MA, Hocking AD, Pitt JI, Poulos PG (2003). Influence of temperature, water activity and pH on growth of some xerophilic fungi. Int J Food Microbiol.

[b25] Golyshina OV, Golyshin PN, Timmis KN, Ferrer M (2006). The ‘pH optimum anomaly’ of intracellular enzymes of *Ferroplasma acidiphilum*. Environ Microbiol.

[b26] Grant WD (2004). Life at low water activity. Philos Trans R Soc Lond, B, Biol Sci.

[b27] Griffin DM (1977). Water potential and wood-decay fungi. Annu Rev Phytopathol.

[b28] Gunde-Cimerman N, Zalar P, de Hoog S, Plemenitas A (2000). Hypersaline waters in salterns – natural ecological niches for halophilic black yeasts. FEMS Microbiol Ecol.

[b29] Hallsworth JE (1998). Ethanol-induced water stress in yeast. J Ferment Bioeng.

[b30] Hallsworth JE, Magan N (1994a). Effect of carbohydrate type and concentration on polyhydroxy alcohol and trehalose content of conidia of 3 entomopathogenic fungi. Microbiology.

[b31] Hallsworth JE, Magan N (1994b). Improved biological control by changing polyols/ trehalose in conidia of entomopathogens. Brighton Crop Protection Council – Pests and Diseases 1994.

[b32] Hallsworth JE, Magan N (1995). Manipulation of intracellular glycerol and erythritol enhances germination of conidia at low water availability. Microbiology.

[b33] Hallsworth JE, Magan N (1996). Culture age, temperature and pH affect the polyol and trehalose contents of fungal propagules. Appl Environ Microbiol.

[b34] Hallsworth JE, Magan N (1999). Water and temperature relations of growth of the entomogenous fungi *Beauveria bassiana*, *Metarhizium anisopliae*, and *Paecilomyces farinosus*. J Invertebr Pathol.

[b35] Hallsworth JE, Nomura Y (1999). A simple method to determine the water activity of ethanol-containing samples. Biotechnol Bioeng.

[b36] Hallsworth JE, Heim S, Timmis KN (2003a). Chaotropic solutes cause water stress in *Pseudomonas putida*. Environ Microbiol.

[b37] Hallsworth JE, Prior BA, Nomura Y, Iwahara M, Timmis KN (2003b). Compatible solutes protect against chaotrope (ethanol) -induced, nonosmotic water stress. Appl Environ Microbiol.

[b38] Hallsworth JE, Yakimov MM, Golyshin PN, Gillion JLM, D'Auria G, Alves FDL (2007). Limits of life in MgCl__2__-containing environments: chaotropicity defines the window. Environ Microbiol.

[b39] Hamaguchi K, Geiduschek EP (1962). The effects of electrolytes on the stability of the desoxyribonucleate helix. J Am Chem Soc.

[b40] van der Heijden MGA, Bardgett RD, van Straalen NM (2008). The unseen majority: soil microbes as drivers of plant diversity and productivity in terrestrial ecosystems. Ecol Lett.

[b41] Hocking AD, Jennings DH (1993). Responses of xerophilic fungi to changes in water activity. Stress Tolerance of Fungi..

[b42] Hocking AD, Pitt JI (1980). Dichloran-glycerol medium for enumeration of xerophilic fungi from low-moisture foods. Appl Environ Microbiol.

[b43] Hocking AD, Pitt JI (1988). Two new species of xerophilic fungi and a further record of. Eurotium-Halophilicum Mycologia.

[b44] Hoffland E, Kuyper TW, Wallander H, Plassard C, Gorbushina AA, Haselwandter K (2004). The role of fungi in weathering. Front Ecol Environ.

[b45] Jeffries P, Gianinazzi S, Perotto S, Turnau K, Barea JM (2003). The contribution of arbuscular mycorrhizal fungi in sustainable maintenance of plant health and soil fertility. Biol Fertil Soils.

[b46] de Jong JC, McCormack BJ, Smirnoff N, Talbot NJ (1997). Glycerol generates turgor in rice blast. Nature.

[b47] Kashangura C, Hallsworth JE, Mswaka AY (2006). Phenotypic diversity amongst strains of *Pleurotus sajor-caju*: implications for cultivation in arid environments. Mycol Res.

[b48] Kinderlerer JL (1995). Czapek-Casein 50-Percent Glucose (Czc50g) – a new medium for the identification of foodborne *Chrysosporium* spp. Lett Appl Microbiol.

[b49] Lo Nostro P, Ninham BW, Lo Nostro A, Pesavento G, Fratoni L, Baglioni P (2005). Specific ion effects on the growth rates of *Staphylococcus aureus* and *Pseudomonas aeruginosa*. Phys Biol.

[b50] McCammick EM, Gomase VS, Timson DJ, McGenity TJ, Hallsworth JE, Timmis KN (2009). Water–hydrophobic compound interactions with the microbial cell. Handbook of Hydrocarbon and Lipid Microbiology – Hydrocarbons, Oils and Lipids: Diversity, Properties and Formation.

[b51] Marion GM, Kargel JS (2008). Cold Aqueous Planetary Geochemistry with FREZCHEM: From Modeling to the Search for Life at the Limits.

[b52] Marris E (2008). Water: More crop per drop. Nature.

[b53] Mustard JF, Murchie SL, Pelkey SM, Ehlmann BL, Milliken RE, Grant JA (2008). Hydrated silicate minerals on Mars observed by the Mars reconnaissance orbiter CRISM instrument. Nature.

[b54] Onofri S, Selbmann L, Zucconi L, Pagano S (2004). Antarctic microfungi as models for exobiology. Planet Space Sci.

[b55] Oren A (1983). *Halobacterium sodomense* sp. nov., a Dead Sea Halobacterium with an extremely high magnesium requirement. Int J Syst Bacteriol.

[b56] Pitt JI, Duckworth RB (1975). Xerophilic fungi and the spoilage of foods of plant origin. Water Relations of Foods.

[b57] Pitt JI, Christian JHB (1968). Water relations of xerophilic fungi isolated from prunes. Appl Microbiol.

[b58] Pitt JI, Hocking AD (1977). Influence of solute and hydrogen-ion concentration on water relations of some xerophilic fungi. J Gen Microbiol.

[b59] Ruiz Lozano JM, Azcon R (1995). Hyphal contribution to water uptake in mycorrhizal plants as affected by the fungal species and water status. Physiol Plantarum.

[b60] Rutz BA, Kieft TL (2004). Phylogenetic characterization of dwarf archaea and bacteria from a semiarid soil. Soil Biol Biochem.

[b61] Sachs JN, Woolf TB (2003). Understanding the Hofmeister effect in interactions between chaotropic anions and lipid bilayers: Molecular dynamics simulations. J Am Chem Soc.

[b62] Scott WJ (1957). Water relations of food spoilage microorganisms. Adv Food Res.

[b63] Seager R, Ting MF, Held I, Kushnir Y, Lu J, Vecchi G (2007). Model projections of an imminent transition to a more arid climate in southwestern North America. Science.

[b64] Smith ML, Bruhn JN, Anderson JB (1992). The fungus *Armillaria-bulbosa* is amongst the largest and oldest living organisms. Nature.

[b65] Tamura M, Kawasaki H, Sugiyama J (1999). Identity of the xerophilic species *Aspergillus penicillioides*: Integrated analysis of the genotypic and phenotypic characters. J Gen Appl Microbiol.

[b66] Thomas DSG, Knight M, Wiggs GFS (2005). Remobilization of southern African desert dune systems by twenty-first century global warming. Nature.

[b67] Thomas KC, Hynes SH, Ingledew WM (1994). Effects of particulate materials and osmoprotectants on very-high-gravity ethanolic fermentation by *Saccharomyces cerevisiae*. Appl Environ Microbiol.

[b68] Tosca NJ, Knoll AH, McLennan SM (2008). Water activity and the challenge for life on early Mars. Science.

[b69] Vaupotic T, Plemenitas A (2007). Differential gene expression and Hog1 interaction with osmoresponsive genes in the extremely halotolerant black yeast *Hortaea werneckii*. BMC Genomics.

[b70] Ward N, Fraser CM (2005). How genomics has affected the concept of microbiology. Curr Opin Microbiol.

[b71] Whitman WB, Coleman DC, Wiebe WJ (1998). Prokaryotes: The unseen majority. Proc Natl Acad Sci USA.

[b72] Winston PW, Bates DH (1960). Saturated solutions for the control of humidity in biological research. Ecology.

[b73] Zalar P, de Hoog GS, Schroers H-J, Frank JM, Gunde-Cimerman N (2005). Taxonomy and phylogeny of the xerophilic genus *Wallemia* (Wallemiomycetes and Wallemiales, cl. et ord. nov.). Antonie Van Leeuwenhoek International J Gen Mol Microbiology.

[b74] Zalar P, de Hoog GS, Schroers H-J, Crous PW, Groenewald JZ, Gunde-Cimerman N (2007). Phylogeny and ecology of the ubiquitous saprobe *Cladosporium sphaerospermum*, with descriptions of seven new species from hypersaline environments. Stud Mycol.

[b75] Zhuge J, Fang HY, Wang ZX, Chen DZ, Jin HR, Gu HL (2001). Glycerol production by a novel osmotolerant yeast *Candida glycerinogenes*. Appl Microbiol Biotechnol.

