# Supplementary material for: Limits of life in hostile environments: no barriers to biosphere function?
Source: Environ Microbiol. 2009 Dec;11(12):3292–308. doi: 10.1111/j.1462-2920.2009.02079.x (PMC2810447; doi:10.1111/j.1462-2920.2009.02079.x)
Supplement: Supplementary file 1 [file emi0011-3292-SD1.ppt]

## Slide 1
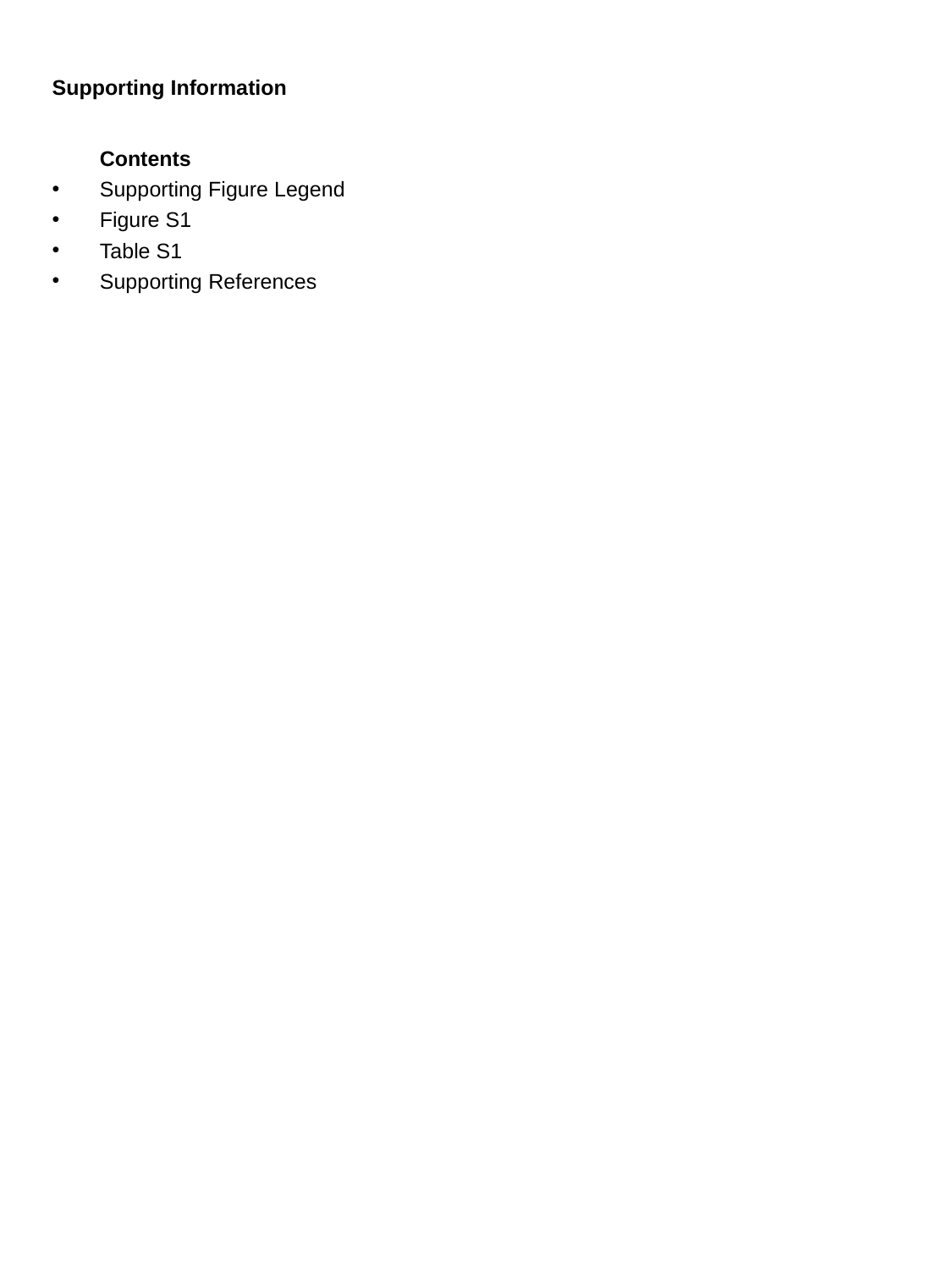

# Supporting Information
Contents
Supporting Figure Legend
Figure S1
Table S1
Supporting References

## Slide 2
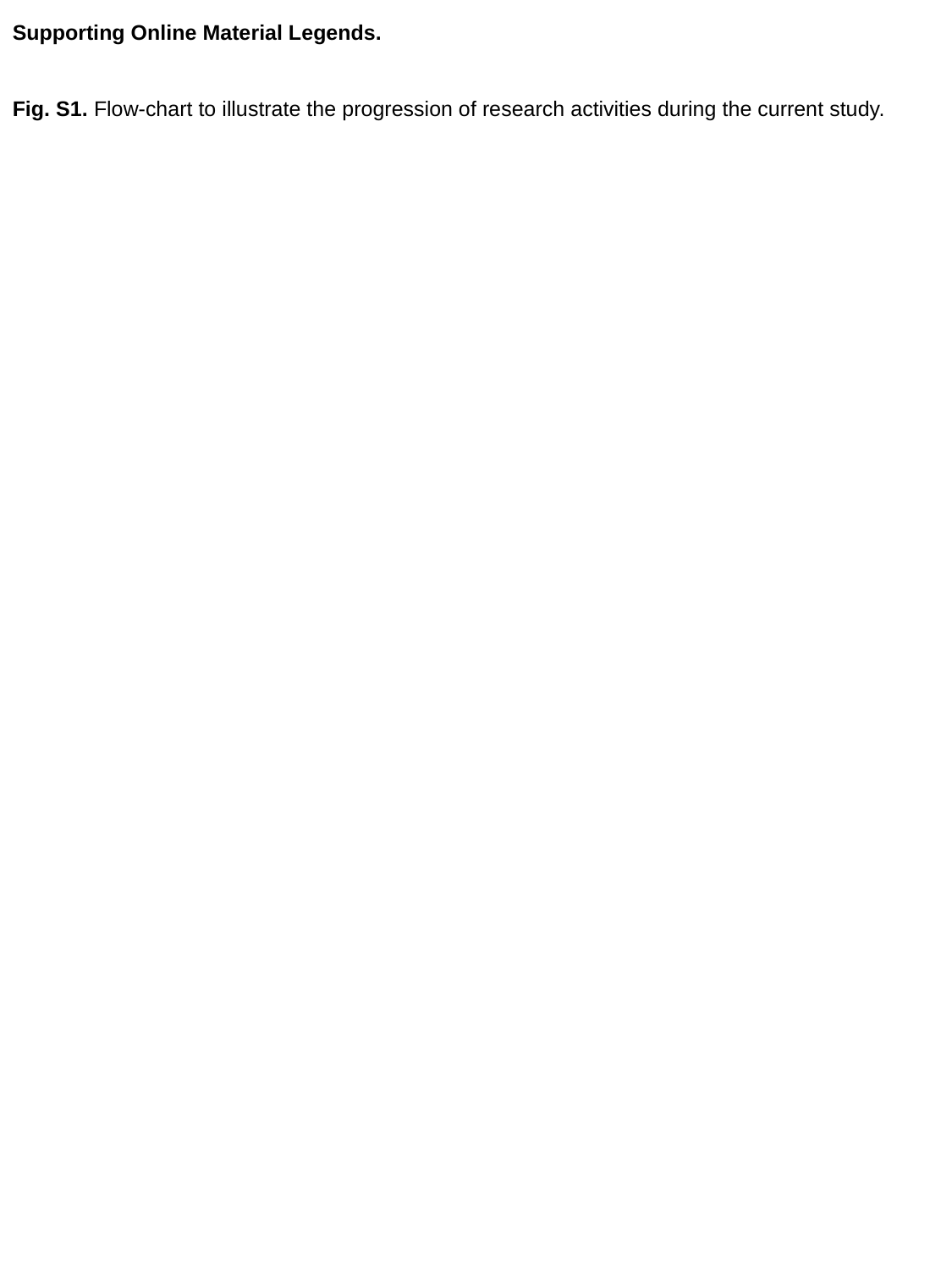

Supporting Online Material Legends.
Fig. S1. Flow-chart to illustrate the progression of research activities during the current study.

## Slide 3
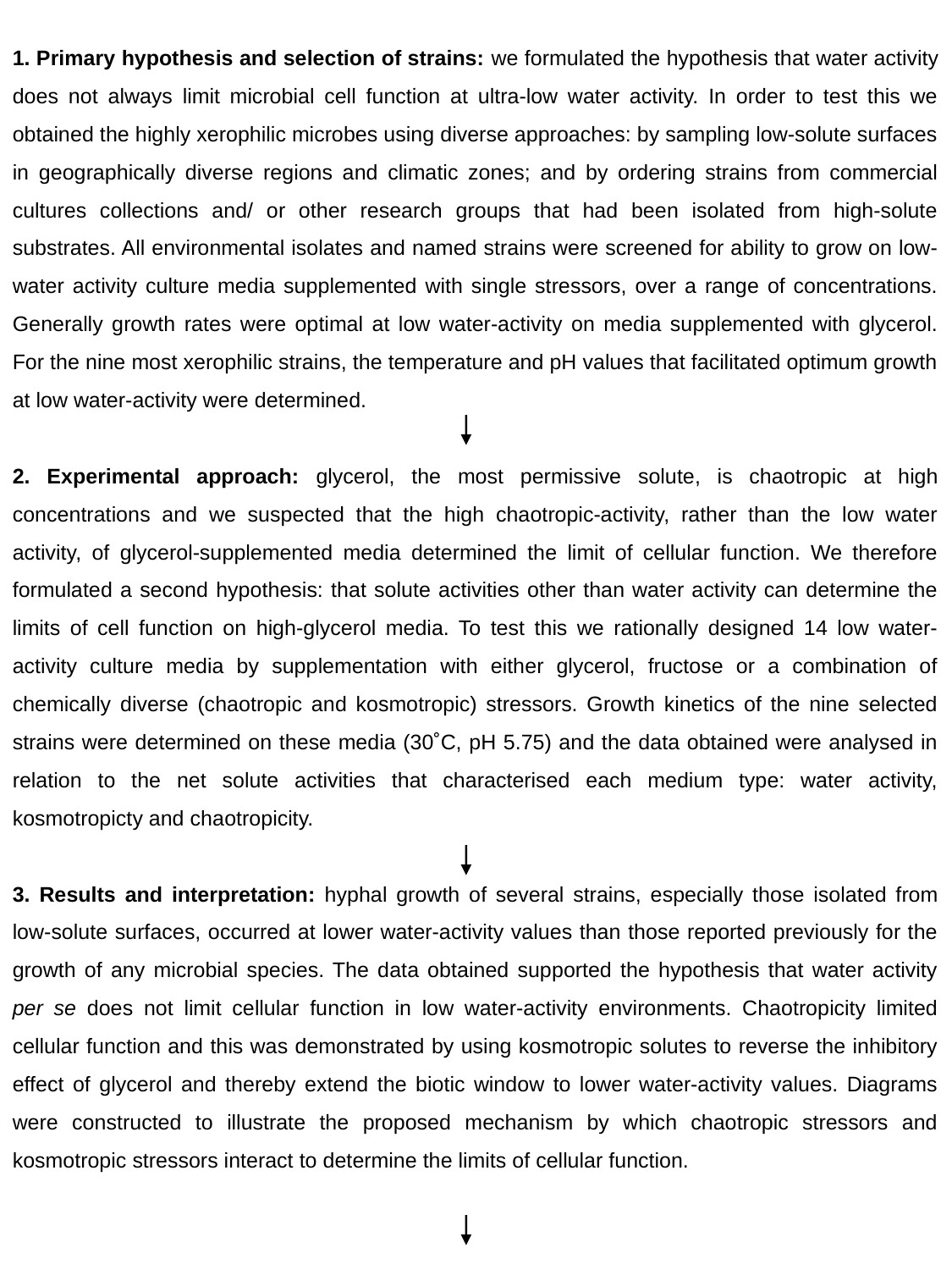

1. Primary hypothesis and selection of strains: we formulated the hypothesis that water activity does not always limit microbial cell function at ultra-low water activity. In order to test this we obtained the highly xerophilic microbes using diverse approaches: by sampling low-solute surfaces in geographically diverse regions and climatic zones; and by ordering strains from commercial cultures collections and/ or other research groups that had been isolated from high-solute substrates. All environmental isolates and named strains were screened for ability to grow on low-water activity culture media supplemented with single stressors, over a range of concentrations. Generally growth rates were optimal at low water-activity on media supplemented with glycerol. For the nine most xerophilic strains, the temperature and pH values that facilitated optimum growth at low water-activity were determined.
2. Experimental approach: glycerol, the most permissive solute, is chaotropic at high concentrations and we suspected that the high chaotropic-activity, rather than the low water activity, of glycerol-supplemented media determined the limit of cellular function. We therefore formulated a second hypothesis: that solute activities other than water activity can determine the limits of cell function on high-glycerol media. To test this we rationally designed 14 low water-activity culture media by supplementation with either glycerol, fructose or a combination of chemically diverse (chaotropic and kosmotropic) stressors. Growth kinetics of the nine selected strains were determined on these media (30˚C, pH 5.75) and the data obtained were analysed in relation to the net solute activities that characterised each medium type: water activity, kosmotropicty and chaotropicity.
3. Results and interpretation: hyphal growth of several strains, especially those isolated from low-solute surfaces, occurred at lower water-activity values than those reported previously for the growth of any microbial species. The data obtained supported the hypothesis that water activity per se does not limit cellular function in low water-activity environments. Chaotropicity limited cellular function and this was demonstrated by using kosmotropic solutes to reverse the inhibitory effect of glycerol and thereby extend the biotic window to lower water-activity values. Diagrams were constructed to illustrate the proposed mechanism by which chaotropic stressors and kosmotropic stressors interact to determine the limits of cellular function.

## Slide 4
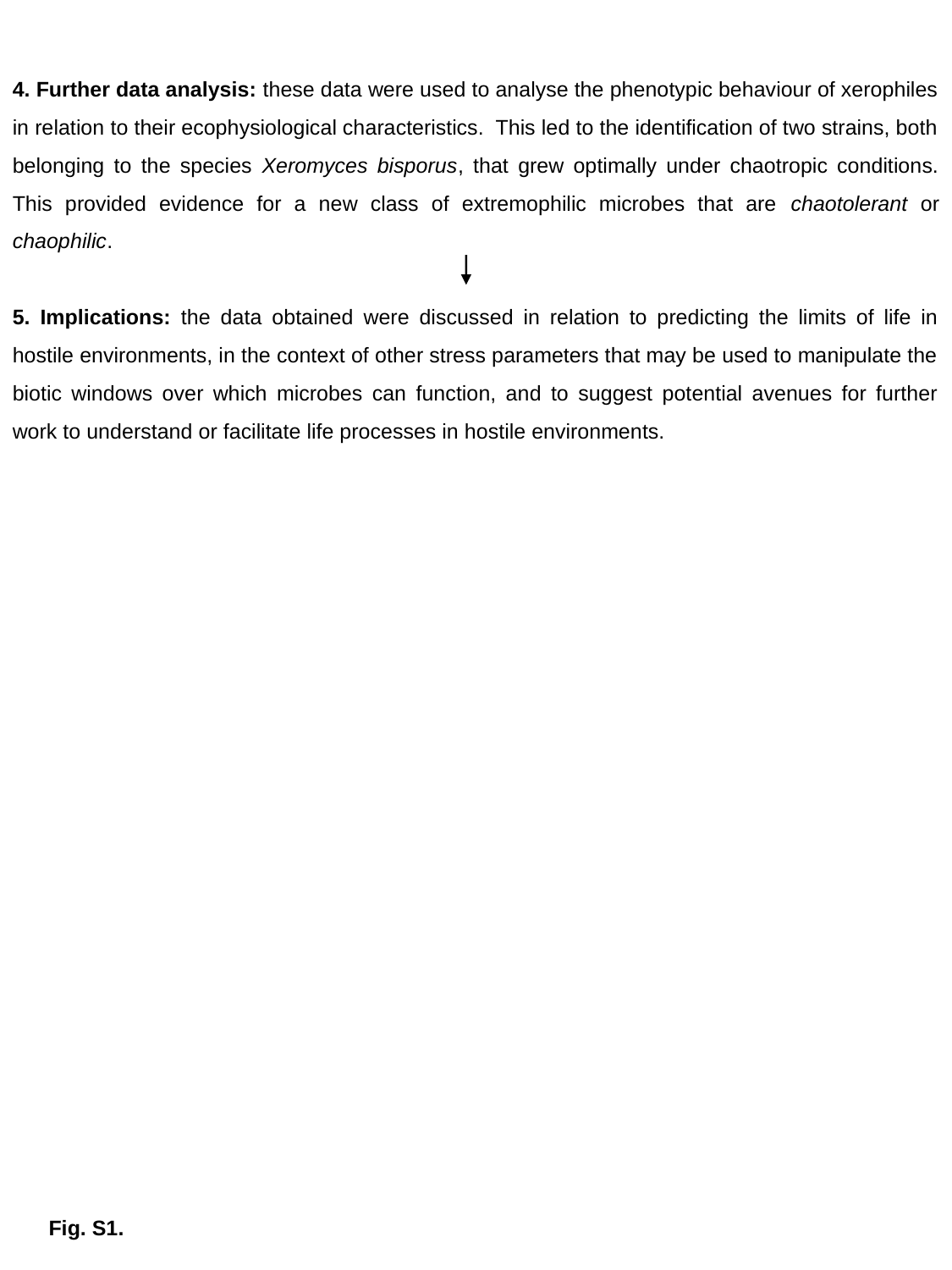

4. Further data analysis: these data were used to analyse the phenotypic behaviour of xerophiles in relation to their ecophysiological characteristics. This led to the identification of two strains, both belonging to the species Xeromyces bisporus, that grew optimally under chaotropic conditions. This provided evidence for a new class of extremophilic microbes that are chaotolerant or chaophilic.
5. Implications: the data obtained were discussed in relation to predicting the limits of life in hostile environments, in the context of other stress parameters that may be used to manipulate the biotic windows over which microbes can function, and to suggest potential avenues for further work to understand or facilitate life processes in hostile environments.
Fig. S1.

## Slide 5
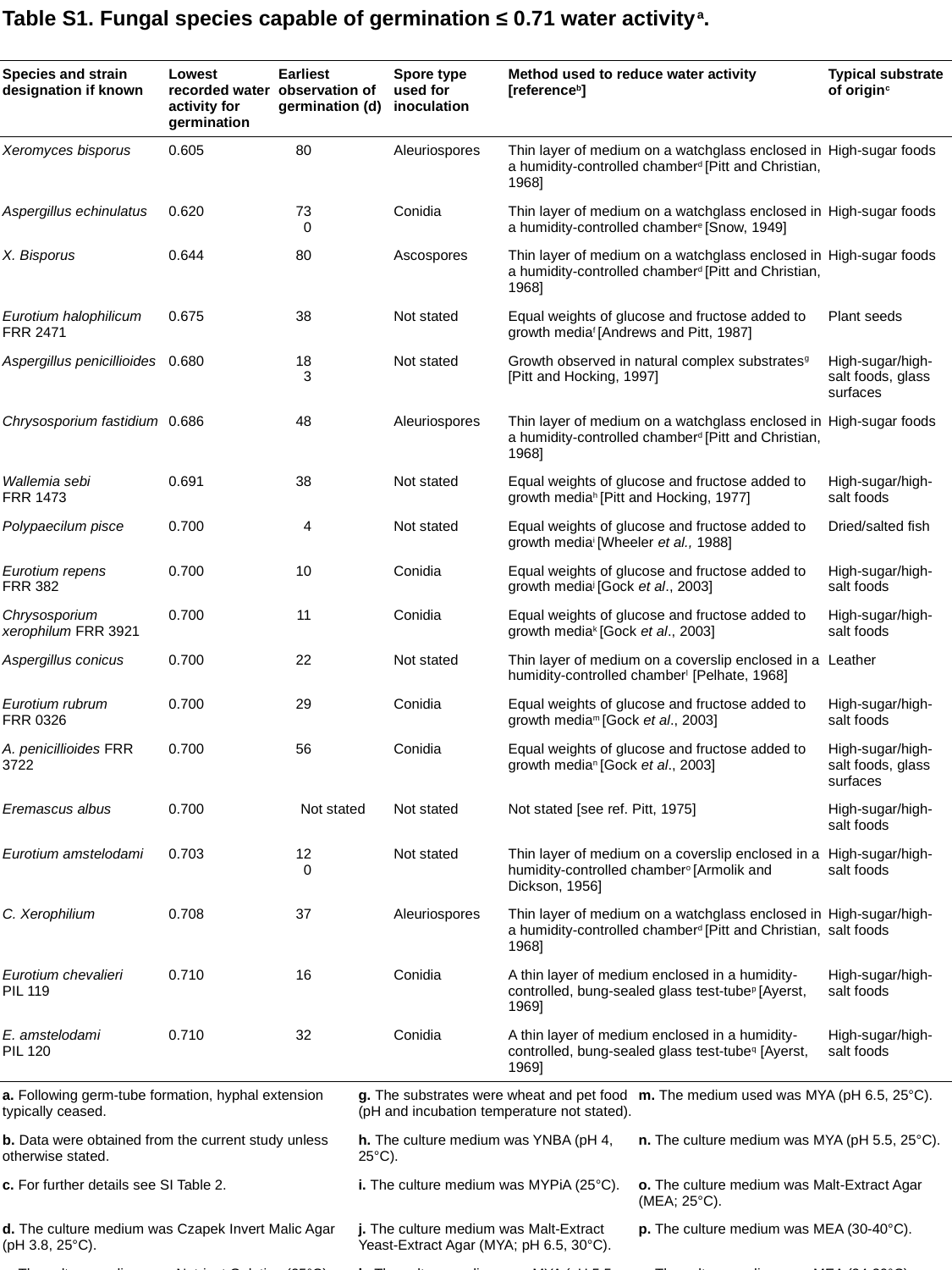

| Table S1. Fungal species capable of germination ≤ 0.71 water activitya. | | | | | | | |
| --- | --- | --- | --- | --- | --- | --- | --- |
| Species and strain designation if known | Lowest recorded water activity for germination | Earliest observation of germination (d) | | Spore type used for inoculation | Method used to reduce water activity [referenceb] | | Typical substrate of originc |
| Xeromyces bisporus | 0.605 | 80 | | Aleuriospores | Thin layer of medium on a watchglass enclosed in a humidity-controlled chamberd [Pitt and Christian, 1968] | | High-sugar foods |
| Aspergillus echinulatus | 0.620 | 730 | | Conidia | Thin layer of medium on a watchglass enclosed in a humidity-controlled chambere [Snow, 1949] | | High-sugar foods |
| X. Bisporus | 0.644 | 80 | | Ascospores | Thin layer of medium on a watchglass enclosed in a humidity-controlled chamberd [Pitt and Christian, 1968] | | High-sugar foods |
| Eurotium halophilicum FRR 2471 | 0.675 | 38 | | Not stated | Equal weights of glucose and fructose added to growth mediaf [Andrews and Pitt, 1987] | | Plant seeds |
| Aspergillus penicillioides | 0.680 | 183 | | Not stated | Growth observed in natural complex substratesg [Pitt and Hocking, 1997] | | High-sugar/high-salt foods, glass surfaces |
| Chrysosporium fastidium | 0.686 | 48 | | Aleuriospores | Thin layer of medium on a watchglass enclosed in a humidity-controlled chamberd [Pitt and Christian, 1968] | | High-sugar foods |
| Wallemia sebi FRR 1473 | 0.691 | 38 | | Not stated | Equal weights of glucose and fructose added to growth mediah [Pitt and Hocking, 1977] | | High-sugar/high-salt foods |
| Polypaecilum pisce | 0.700 | 4 | | Not stated | Equal weights of glucose and fructose added to growth mediai [Wheeler et al., 1988] | | Dried/salted fish |
| Eurotium repens FRR 382 | 0.700 | 10 | | Conidia | Equal weights of glucose and fructose added to growth mediaj [Gock et al., 2003] | | High-sugar/high-salt foods |
| Chrysosporium xerophilum FRR 3921 | 0.700 | 11 | | Conidia | Equal weights of glucose and fructose added to growth mediak [Gock et al., 2003] | | High-sugar/high-salt foods |
| Aspergillus conicus | 0.700 | 22 | | Not stated | Thin layer of medium on a coverslip enclosed in a humidity-controlled chamberl [Pelhate, 1968] | | Leather |
| Eurotium rubrum FRR 0326 | 0.700 | 29 | | Conidia | Equal weights of glucose and fructose added to growth mediam [Gock et al., 2003] | | High-sugar/high-salt foods |
| A. penicillioides FRR 3722 | 0.700 | 56 | | Conidia | Equal weights of glucose and fructose added to growth median [Gock et al., 2003] | | High-sugar/high-salt foods, glass surfaces |
| Eremascus albus | 0.700 | Not stated | | Not stated | Not stated [see ref. Pitt, 1975] | | High-sugar/high-salt foods |
| Eurotium amstelodami | 0.703 | 120 | | Not stated | Thin layer of medium on a coverslip enclosed in a humidity-controlled chambero [Armolik and Dickson, 1956] | | High-sugar/high-salt foods |
| C. Xerophilium | 0.708 | 37 | | Aleuriospores | Thin layer of medium on a watchglass enclosed in a humidity-controlled chamberd [Pitt and Christian, 1968] | | High-sugar/high-salt foods |
| Eurotium chevalieri PIL 119 | 0.710 | 16 | | Conidia | A thin layer of medium enclosed in a humidity-controlled, bung-sealed glass test-tubep [Ayerst, 1969] | | High-sugar/high-salt foods |
| E. amstelodami PIL 120 | 0.710 | 32 | | Conidia | A thin layer of medium enclosed in a humidity-controlled, bung-sealed glass test-tubeq [Ayerst, 1969] | | High-sugar/high-salt foods |
| a. Following germ-tube formation, hyphal extension typically ceased. | | | g. The substrates were wheat and pet food (pH and incubation temperature not stated). | | | m. The medium used was MYA (pH 6.5, 25°C). | |
| b. Data were obtained from the current study unless otherwise stated. | | | h. The culture medium was YNBA (pH 4, 25°C). | | | n. The culture medium was MYA (pH 5.5, 25°C). | |
| c. For further details see SI Table 2. | | | i. The culture medium was MYPiA (25°C). | | | o. The culture medium was Malt-Extract Agar (MEA; 25°C). | |
| d. The culture medium was Czapek Invert Malic Agar (pH 3.8, 25°C). | | | j. The culture medium was Malt-Extract Yeast-Extract Agar (MYA; pH 6.5, 30°C). | | | p. The culture medium was MEA (30-40°C). | |
| e. The culture medium was Nutrient Gelatine (25°C). | | | k. The culture medium was MYA (pH 5.5, 30°C). | | | q. The culture medium was MEA (24-30°C). | |
| f. The culture medium was Yeast Nitrogen-Base Agar (YNBA; 25°C). | | | l. The culture medium was MYA (22°C) | | | | |

## Slide 6
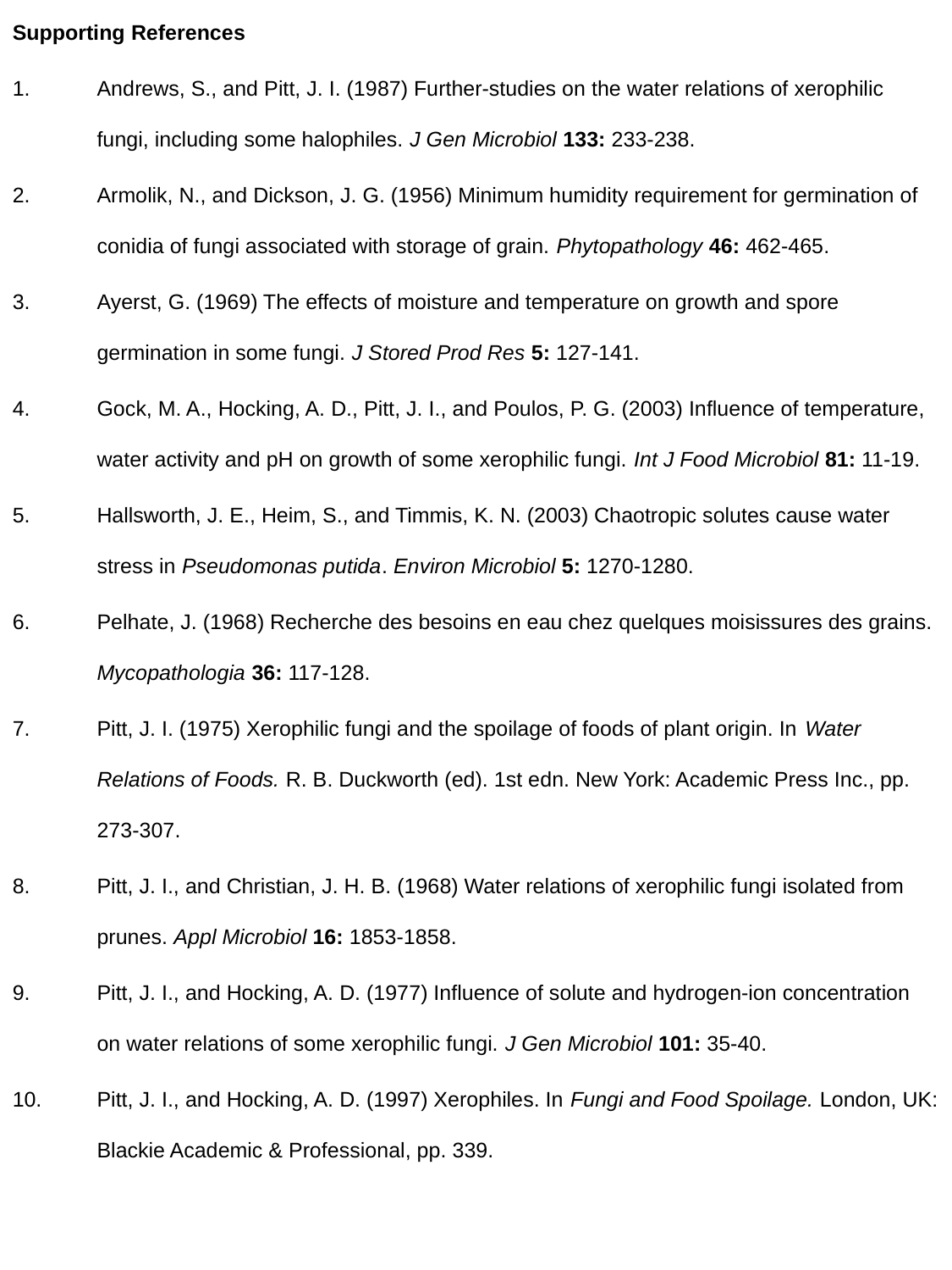

Supporting References
Andrews, S., and Pitt, J. I. (1987) Further-studies on the water relations of xerophilic fungi, including some halophiles. J Gen Microbiol 133: 233-238.
Armolik, N., and Dickson, J. G. (1956) Minimum humidity requirement for germination of conidia of fungi associated with storage of grain. Phytopathology 46: 462-465.
Ayerst, G. (1969) The effects of moisture and temperature on growth and spore germination in some fungi. J Stored Prod Res 5: 127-141.
Gock, M. A., Hocking, A. D., Pitt, J. I., and Poulos, P. G. (2003) Influence of temperature, water activity and pH on growth of some xerophilic fungi. Int J Food Microbiol 81: 11-19.
Hallsworth, J. E., Heim, S., and Timmis, K. N. (2003) Chaotropic solutes cause water stress in Pseudomonas putida. Environ Microbiol 5: 1270-1280.
Pelhate, J. (1968) Recherche des besoins en eau chez quelques moisissures des grains. Mycopathologia 36: 117-128.
Pitt, J. I. (1975) Xerophilic fungi and the spoilage of foods of plant origin. In Water Relations of Foods. R. B. Duckworth (ed). 1st edn. New York: Academic Press Inc., pp. 273-307.
Pitt, J. I., and Christian, J. H. B. (1968) Water relations of xerophilic fungi isolated from prunes. Appl Microbiol 16: 1853-1858.
Pitt, J. I., and Hocking, A. D. (1977) Influence of solute and hydrogen-ion concentration on water relations of some xerophilic fungi. J Gen Microbiol 101: 35-40.
Pitt, J. I., and Hocking, A. D. (1997) Xerophiles. In Fungi and Food Spoilage. London, UK: Blackie Academic & Professional, pp. 339.

## Slide 7
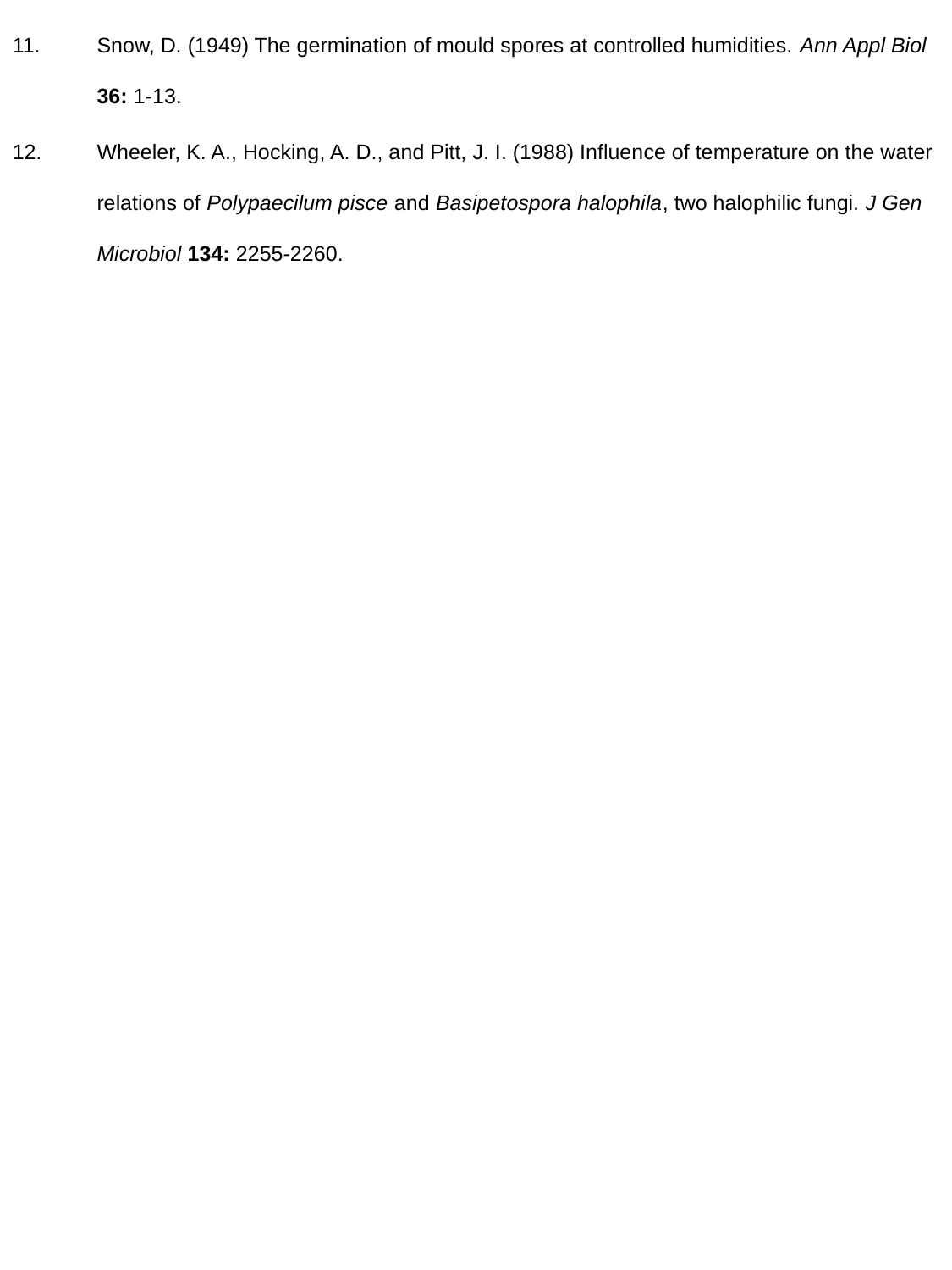

Snow, D. (1949) The germination of mould spores at controlled humidities. Ann Appl Biol 36: 1-13.
Wheeler, K. A., Hocking, A. D., and Pitt, J. I. (1988) Influence of temperature on the water relations of Polypaecilum pisce and Basipetospora halophila, two halophilic fungi. J Gen Microbiol 134: 2255-2260.
